# Haplotype-resolved genome of diploid ginger (*Zingiber officinale*) and its unique gingerol biosynthetic pathway

**DOI:** 10.1038/s41438-021-00627-7

**Published:** 2021-08-05

**Authors:** Hong-Lei Li, Lin Wu, Zhaoming Dong, Yusong Jiang, Sanjie Jiang, Haitao Xing, Qiang Li, Guocheng Liu, Shuming Tian, Zhangyan Wu, Bin Wu, Zhexin Li, Ping Zhao, Yan Zhang, Jianmin Tang, Jiabao Xu, Ke Huang, Xia Liu, Wenlin Zhang, Qinhong Liao, Yun Ren, Xinzheng Huang, Qingzhi Li, Chengyong Li, Yi Wang, Baskaran Xavier-Ravi, Honghai Li, Yang Liu, Tao Wan, Qinhu Liu, Yong Zou, Jianbo Jian, Qingyou Xia, Yiqing Liu

**Affiliations:** 1 College of Landscape Architecture and Life Science/Institute of Special Plants, Chongqing University of Arts and Sciences, Yongchuan, Chongqing China; 2 Engineering Research Center for Special Plant Seedlings of Chongqing, Chongqing University of Arts and Sciences, Yongchuan, Chongqing China; 3 State Key Laboratory of Silkworm Genome Biology, Biological Science Research Center, Southwest University, Beibei, Chongqing China; 4 BGI Genomics, BGI-Shenzhen, Shenzhen, Guangdong China; 5 College of Biology and Food Engineering, Chongqign Three Gorges University, Wanzhou, Chongqing China; 6 Department of Entomology and MOAKey Lab of Pest Monitoring and Green Management, College of Plant Protection, China Agricultural University, Haidian, Beijing China; 7 Jinan Second Agricultural Science Research Institute, Jinan, Shandong China; 8 Savari Research Foundation, Mela Ilandai Kulam, Tamil Nadu India; 9 Institute of Advanced Interdisciplinary Studies, Chongqing University, Chongqing, China; 10 Fairy Lake Botanical Garden and Chinese Academy of Sciences, Shenzhen, Guangdong China; 11 Ningyang Science and Technology Bureau, Taian, Shandong China; 12 College of Horticulture and Gardening, Yangtze University, Jingzhou, Hubei China

**Keywords:** Genome, Genomics

## Abstract

Ginger (*Zingiber officinale*), the type species of Zingiberaceae, is one of the most widespread medicinal plants and spices. Here, we report a high-quality, chromosome-scale reference genome of ginger ‘Zhugen’, a traditionally cultivated ginger in Southwest China used as a fresh vegetable, assembled from PacBio long reads, Illumina short reads, and high-throughput chromosome conformation capture (Hi-C) reads. The ginger genome was phased into two haplotypes, haplotype 1 (1.53 Gb with a contig N50 of 4.68 M) and haplotype 0 (1.51 Gb with a contig N50 of 5.28 M). Homologous ginger chromosomes maintained excellent gene pair collinearity. In 17,226 pairs of allelic genes, 11.9% exhibited differential expression between alleles. Based on the results of ginger genome sequencing, transcriptome analysis, and metabolomic analysis, we proposed a backbone biosynthetic pathway of gingerol analogs, which consists of 12 enzymatic gene families, *PAL*, *C4H*, *4CL*, *CST*, *C3’H*, *C3OMT*, *CCOMT*, *CSE*, *PKS*, *AOR*, *DHN*, and *DHT*. These analyses also identified the likely transcription factor networks that regulate the synthesis of gingerol analogs. Overall, this study serves as an excellent resource for further research on ginger biology and breeding, lays a foundation for a better understanding of ginger evolution, and presents an intact biosynthetic pathway for species-specific gingerol biosynthesis.

## Introduction

Ginger (*Zingiber officinale*) is an herbaceous perennial from the Zingiberaceae family that has great importance as a spice^1^. It is one of the most widely cultivated medicinal crops and one of the best-known nonprescription drugs in the traditional medicinal systems of many countries^2^. Ginger is grown in more than 39 countries worldwide. China and India are the top two ginger producers, and the history of their cultivation in these regions can be traced back over 2000 years. According to data from the FAO, global ginger production in 2019 was 4.08 million tons and had significant economic value in world trade.

More than 60 bioactive compounds have been studied in ginger, including volatile oils, gingerol and diphenyl heptane, free amino acids, starch, resin-like substances, and others^3,4^. In particular, compounds such as gingerols, gingerdiols, zingerone, paradols, and shogaol have been studied for their potential medicinal properties. Based on their pharmacological properties, gingerols are considered to be the most important medicinal compounds in ginger^5^. They consist of 4-, 6-, 7-, 8-, and 10-gingerol structural analogs, although they are thermally labile and can quickly be transformed to shogaols at high temperatures^6,7^. The concentration of 6-gingerol is higher than that of other gingerols in ginger rhizomes, and it is recognized as the major compound responsible for ginger’s pungency. 6-Gingerol also plays an important role in the suppression of hyperproliferation and inflammation, and it inhibits carcinogenesis, as well as subsequent metastasis^8,9^.

Zingiberaceae contains numerous species that are economically valuable as spices, perfumes, and ornamental plants^10^; nonetheless, no whole-genome assemblies are currently available for this family. Within *Zingiber*, the type genus of the ginger family, only the chloroplast genome has been assembled to date, and this lack of genomic resources severely impedes our understanding of ginger genome evolution and gingerol biosynthesis. Here, we report a high-quality, haplotype-resolved chromosome-level genome assembly for cultivated ginger. We also analyzed ginger metabolites and constructed a backbone biosynthetic pathway for gingerol analogs. The genomic resources provided here will be valuable for understanding the unique characteristics of ginger and will promote further biological and agronomic analyses of *Zingiberaceae* species.

## Results

### Genome sequencing, assembly, and annotation

*Zingiber officinale* ‘Zhugen’ (2n = 2x = 22), a traditionally cultivated ginger in Southwest China used as a fresh vegetable, was used for whole-genome sequencing (Supplementary Fig. S1). A total of 369.51 Gb of clean Illumina short-read data (232.4× coverage), 285.81 Gb of PacBio long-read data (179.8× coverage), and 563.16 Gb of Illumina-sequenced Hi-C data were generated (Supplementary Tables S1–3). We evaluated the ginger genome size by k-mer analysis using 64× input data, and the results showed that the ginger genome was approximately 1.59 Gb in size with 3.6% heterozygosity (Supplementary Fig. S2 and Supplementary Table S4). The de novo assembly of genome contigs was performed with Falcon, and the parameter ‘Falcon phase’ was applied for phasing. Contigs were then polished with Arrow and corrected with Pilon (Supplementary Fig. S3). The resulting sequences were phased into two haplotypes named ‘haplotype 1’ and ‘haplotype 0’ (Table 1). Hi-C reads were used to build the 11 pseudochromosomes, and the Hi-C map was validated to show that low-level interactions occurred between rather than within pseudochromosomes, indicating that our chromosome-level anchoring was of high quality and reliable (Supplementary Figs. S4–S6). In total, approximately 98.11% of sequences were anchored onto pseudochromosomes in the two haplotypes (Supplementary Table S6). The genome size of the final assembly for haplotype 1 was 1.53 Gb with 669 contigs (N50 of 4.68 Mb) (Table 1, Supplementary Tables S5 and S6). The genome size of haplotype 0 was 1.51 Gb with 636 contigs (N50 of 5.28 Mb) (Table 1, Supplementary Table S5 and S6). The average GC content of the ginger genome was 39.20%, which is higher than that of banana (*Musa acuminata*, 38.87%; *M. balbisiana*, 38.02%) and lower than that of sorghum (*Sorghum bicolor*, 43.75%) and rice (*Oryza sativa*, 43.57%) (Table 1, Supplementary Fig. S7, Supplementary Table S7). We evaluated the quality of the assembly using Benchmarking Universal Single-Copy Orthologs (BUSCO). Haplotype 1 showed over 94.4% coverage of the embryophyte orthologous gene set, whereas haplotype 0 showed only 93.5% coverage (Supplementary Table S8). The LAI scores of both haplotypes were generally above 10, with an average score of 15 (Supplementary Fig. S8). Furthermore, 94.84% and 93.93% of the expressed transcripts from the ginger RNA-seq dataset were covered in haplotypes 1 and 0, respectively (Supplementary Table S9). Together, these results highlight the high quality of the ginger genome assembly.

**Table 1:** Statistics of the ginger genome

Chromosome number (2n)		2n = 2x = 22	
Estimate of genome size		1,593,035,063 bp	
		Haplotype 1	Haplotype 0
Contig assembly	Total number of contigs	669	636
	Assembly size	1,526,395,517	1,504,782,856
	N50	4,675,000	5,281,000
	N90	1,486,624	1,602,234
	Largest contig	26,686,000	20,644,377
Scaffold assembly	Total number of scaffolds	11	11
	Assembly size	1,527,053,517	1,505,407,856
	N50	141,499,028	142,996,746
	N90	97,488,358	99,672,939
	Largest scaffold	179,820,657	197,841,224
Annotation	GC content	39.20%	39.20%
	Repeat content	56.90%	56.70%
	Number of protein-coding genes	39,217	38,090
	Average length of protein-coding genes	5031	5028

**Fig. 1:**
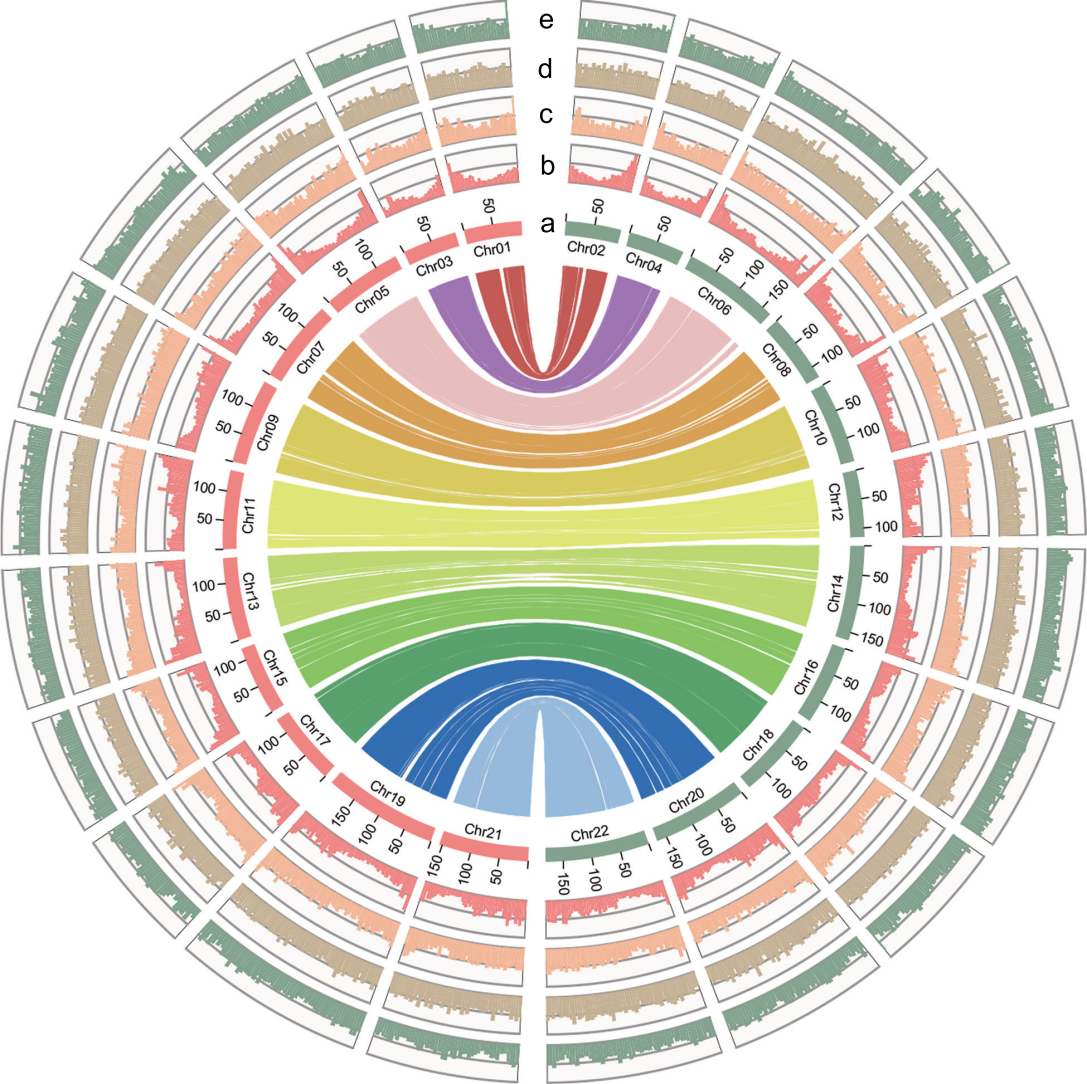
Characteristics of the ginger genome. Distribution of genomic features of the ginger haplotype-resolved genome for haplotype 1 (green) and haplotype 0 (red). From inside to outside: (a) chromosome number, (b) gene density (0–220), (c) SSR density (0–0.002), (d) LTR density (0–0.18), and (e) GC content (0.37–0.42). The links in the center connect syntenic gene blocks that were detected using MCScanX. Chr, chromosome

In addition, 39,217 protein-coding genes were identified in ginger haplotype 1 with an average gene length of 5031 bp, whereas 38,090 protein-coding genes were identified in haplotype 0 with an average gene length of 5028 bp (Supplementary Table S10). Although the gene length, coding sequence (CDS) length, exon length, and intron length were comparable across all tested genomes, the gene number in ginger was larger than that in most of the species whose reference genome sequences have been reported (Supplementary Table S10). In haplotype 0, there were 1958 missing genes, with 486 pseudogenes, 291 fragmented genes, and 1181 genes lost, whereas there were 1620 missing genes, with 268 pseudogenes, 176 fragmented genes, and 1176 genes lost, in haplotype 1. Functional annotation showed that 85.80% and 86.24% of the proteins encoded by genes in haplotype 1 and haplotype 0 matched known proteins in public databases (Supplementary Table S11). Furthermore, BUSCO analysis showed that 88.2% and 87.3% of the predicted genes had full-length sequence information in haplotype 1 and haplotype 0 (Supplementary Table S12). In addition, we mapped the gene characteristics onto the two ginger genome haplotypes (Fig. 1). Unless otherwise specified, haplotype 1 was used for subsequent analyses.

### Haplotype comparison

**Fig. 2:**
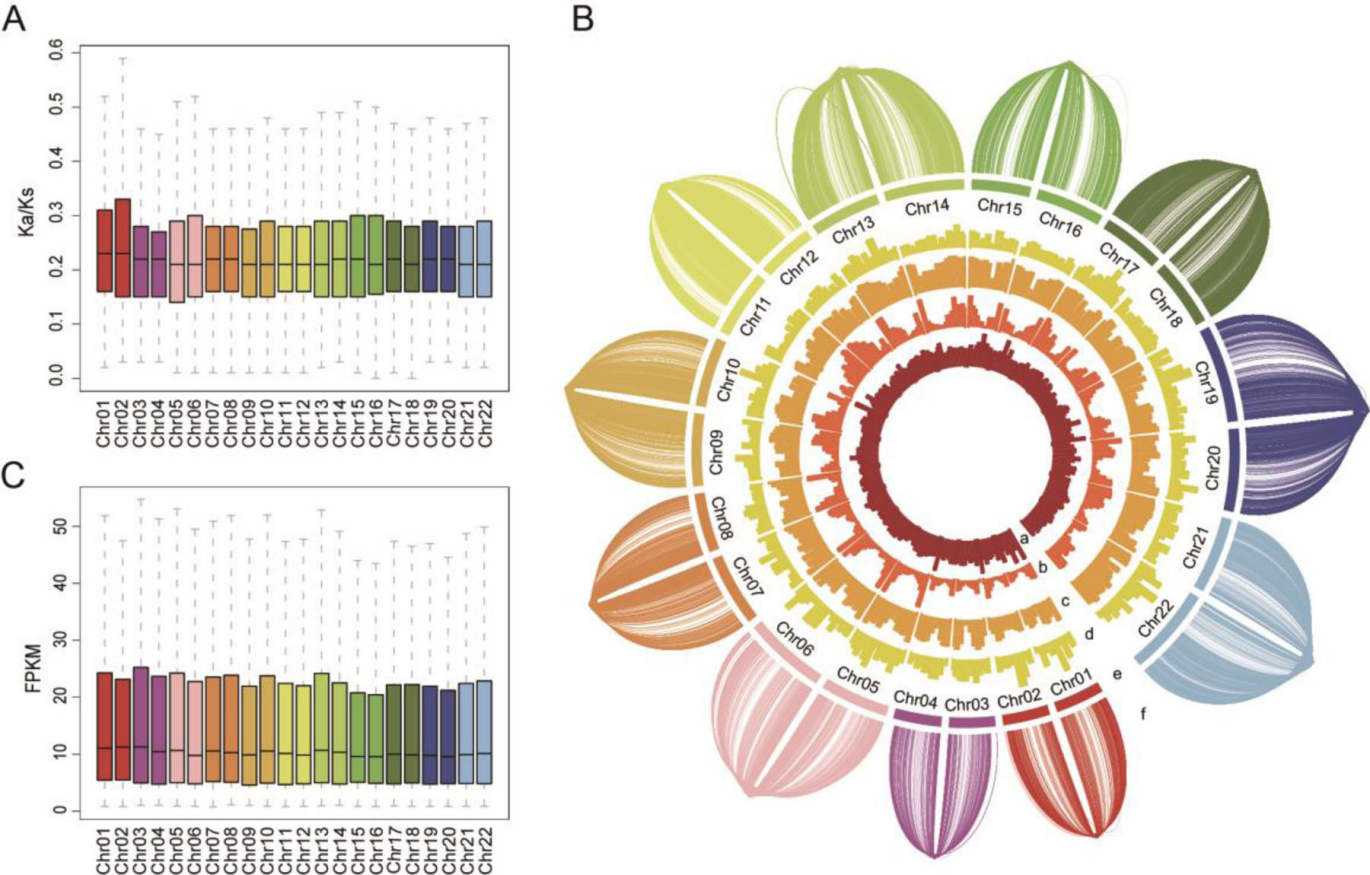
Comparisons between allelic genes of the two ginger haplotypes. **A** The Ka/Ks value of individual chromosomes from both haplotypes. **B** Distribution of allelic gene features of ginger haplotype 1 and haplotype 0. From inside to outside: (a) ratio of heterozygosity (1–5%), (b) number of allelic genes (0–300), (c) ratio of heterozygous genes (40–100%), (d) ratio of DEL (0-15%), (e) chromosome number, and (f) allelic gene links. **C** Box plot of the FPKM value of allelic genes in paired chromosomes of the two haplotypes. The expression data were from ginger stem samples with three biological replicates across five developmental stages

The set of PacBio reads was used to validate the two final haplotypes. There was a 97.95% overlap between the PacBio reads and haplotype 1 and a 98.1% overlap with haplotype 0, indicating that the phasing was precise (Supplementary Fig. S9, Supplementary Table S13). Heterozygosity between the two haplotypes was 3.78%, which is consistent with the k-mer analysis (Supplementary Table S14). Single-copy genes between the two haplotypes were characterized and demonstrated equal distribution (Supplementary Fig. S10). The Ka/Ks ratios of single-copy genes from both haplotypes were consistent, implying that the two haplotypes experienced similar selection pressure during the domestication history of ginger (Fig. 2A). Furthermore, 57 major collinear blocks (with 12 inversions) were identified between the two haplotypes by synteny analysis (Supplementary Figs. S11 and S12, Supplementary Table S15). The raw reads mapped around the reversed regions, especially the breakpoints, support the existence of chromosome inversions in ginger (Supplementary Fig. S13), which were consistent with previous karyotype analysis^11–13^. In total, 55,635 genes (72.0% of all annotated genes) were identified as homologs of the two haplotypes (Supplementary Table S16). The features of 17,226 allelic gene pairs from the two haplotypes were characterized, and most of the features showed similar distribution patterns (Fig. 2B). Consistently, we found that the expression levels of these allelic genes did not differ significantly between haplotypes (Fig. 2C, Supplementary Fig. S14). Interestingly, 2055 gene pairs (11.9%) exhibited differential expression between two alleles, and these differentially expressed loci were mainly enriched in metabolic pathways (Supplementary Figs. S15 and S16).

### Genome evolution

**Fig. 3:**
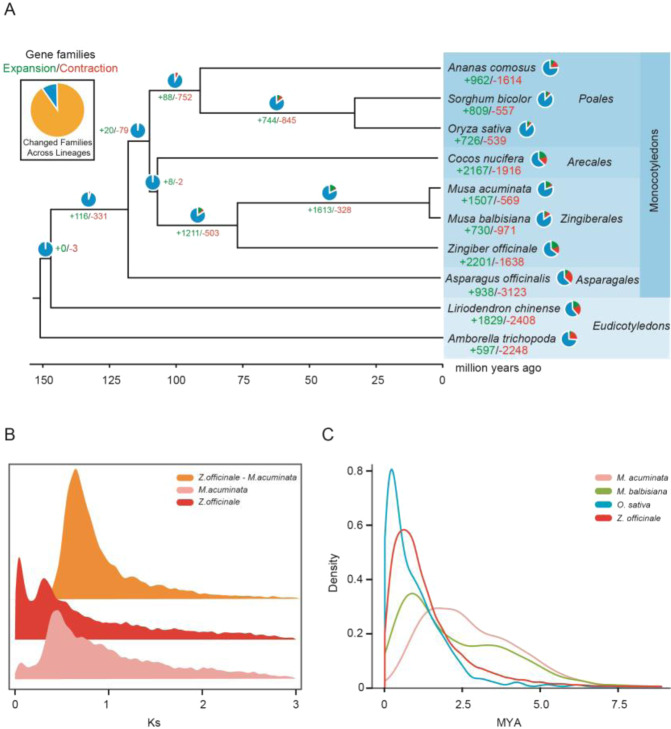
Comparative genomic analysis underlying ginger genome evolution. **A** Phylogenetic relationship and gene family expansion and contraction. The divergence times of ginger were estimated using the topology obtained from the phylogenomic analysis. **B** Density distributions of Ks between syntenic gene pairs and cross-comparison of *M. acuminata* and *Z. officinale*. **C** LTR differentiation time in four plants, *O. sativa*, *M. acuminata*, *M. balbisiana*, and *Z. officinale* (MYA, Million years ago)

To gain insights into the evolution of the ginger genome, we compared the ginger genome with that of nine other plant species: *Amborella trichopoda*, *Ananas comosus*, *Asparagus officinalis*, *Cocos nucifera*, *Liriodendron chinense*, *M. acuminata*, *M. balbisiana*, *O. sativa*, and *S. bicolor* (Supplementary Table S17). In total, 1112 single-copy homologous genes from these 10 species were identified and used for phylogenetic analysis (Fig. 3A and Supplementary Fig. S17). Based on the known divergence times of angiosperms, monocots, Gramineae, Zingiberales, and Zingiberaceae, ginger separated from the Musaceae approximately 76.4 million years ago (MYA) (Fig. 3A). Following this divergence, 1098 gene families showed expansion in ginger, and 20 gene families showed contraction (*P* ≤ 0.01, Supplementary Tables S18 and S19). KEGG analysis suggested that these gene families exhibited several enriched functions (Supplementary Tables S20 and S21). Notably, genes in the expanded families were significantly enriched in metabolic pathways and the biosynthesis of secondary metabolites (Supplementary Fig. 18), whereas genes in the contracted families were mainly enriched in the plant-pathogen interaction pathway (Supplementary Fig. 19).

Distributions of synonymous substitutions (Ks) within genes in syntenic blocks, distribution of transversions at fourfold degenerate sites (4dTv) and genomic synteny analyses indicated that a recent whole-genome duplication (WGD) event occurred in the evolutionary history of *M. acuminata* and *Z. officinale* (Fig. 3B, Supplementary Figs. S20 and S21, Supplementary Table S22). De novo prediction and comparison of the homologs in RepBase indicated that over 63.62% of the ginger genome consisted of transposable elements (TEs) (Supplementary Table S23). Through statistical classification of TEs, we found that the most abundant TEs were long terminal repeats (LTRs). These LTRs occupied up to 61.06% of the genome in the two haplotypes (Supplementary Table S24). LTR sequences with more than five functional domains were selected and used to calculate the differentiation times in four plant species. LTRs in bananas showed an earlier expansion than LTRs in ginger (Fig. 3C). Compared with other plants, the large-scale distribution and activity of LTRs in ginger may be one of the most important reasons for its large genome size.

### Gingerol biosynthesis pathway

**Fig. 4:**
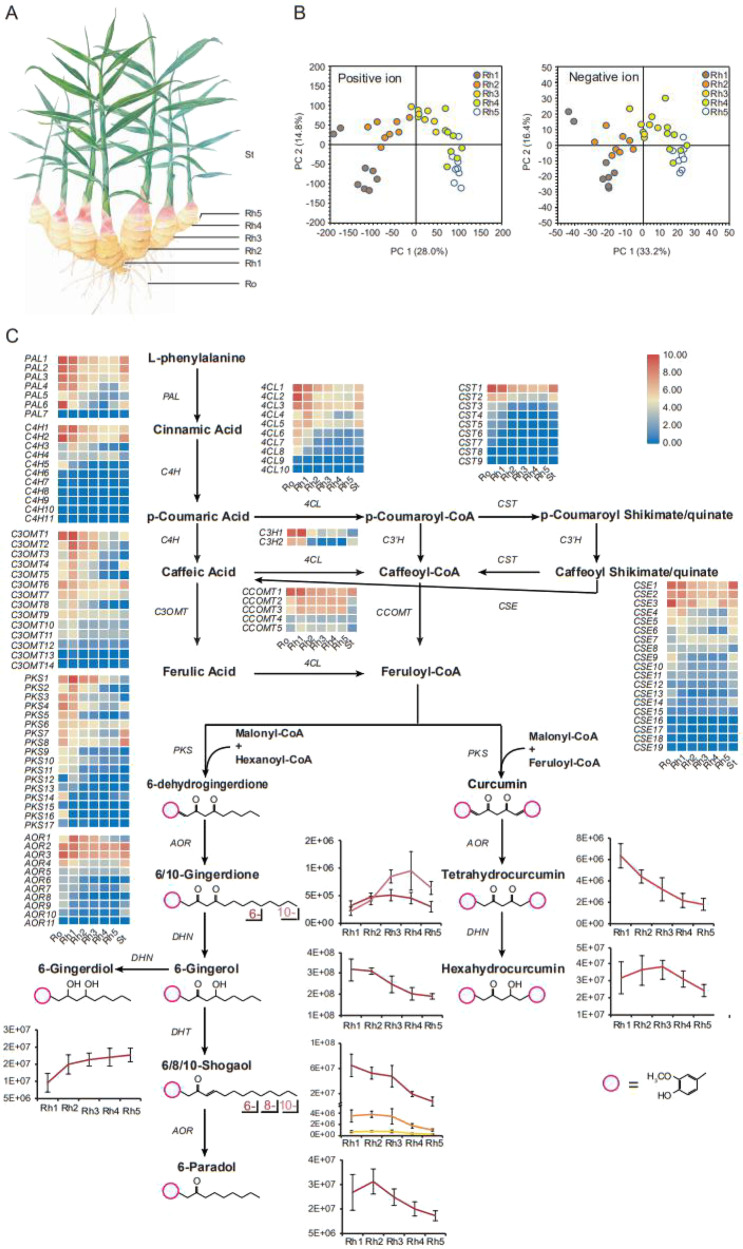
Metabolites in ginger rhizomes and gingerol biosynthesis. **A** Representative graph of ginger showing the root and five rhizome developmental stages was used in sample collection. **B** Principal component analysis (PCA) of ginger rhizome metabolites identified in positive and negative ion modes. Eight biological replicates were performed for each developmental stage. **C** Schematic representation of backbone pathways of gingerol biosynthesis and the expression of key genes. The heatmap shows the level of gene expression in different tissues from red (higher expression) to blue (lower expression). The gene names are given on the left, and the tissue names are given at the bottom. The genes include phenylalanine ammonia lyase (*PAL*), cinnamate 4-hydroxylase (*C4H*), 4-coumarate-CoA ligase (*4CL*), *p*-coumaroyl shikimate transferase (*CST*), *p*-coumaroyl 5-O-quinate/shikimate 3’-hydroxylase (*C3’H*), caffeoylshikimate esterase (*CSE*), caffeic acid 3-O-methyltransferase (*C3OMT*), caffeoyl-CoA O-methyltransferase (*CCOMT*), polyketide synthase (*PKS*), NADPH-dependent alkanal/one oxidoreductase (*AOR*), dehydrogenase (*DHN*), and dehydratase (*DHT*). The line graph shows changes in the metabolite contents at different rhizome developmental stages. The vertical axis represents metabolite abundance

UHPLC-MS/MS was performed to determine the active compounds in ginger rhizomes at five developmental stages. A total of 400 positive and 39 negative ionization compounds were identified in ginger rhizomes (Fig. 4A, B, Supplementary Tables S25 and S26). These metabolites were mainly categorized as secondary metabolites, amino acids, lipids, nucleotides, organic acids, and vitamins (Supplementary Tables S25 and S26). The levels of most amino acids and half of the lipids increased from the mature rhizome (Rh1) to the newly developed rhizome (Rh5), whereas the levels of most organic acids, nucleotides, vitamins and secondary metabolites tended to decrease (Supplementary Fig. S22). The contents of 10 gingerol analogs were also evaluated, including 6-gingerol, 6/10-gingerdione, 6-gingerdiol, 6/8/10-shogaol, 6-paradol, tetrahydrocurcumin, and hexahydrocurcumin. Because gingerol analogs with two aromatic rings are termed curcuminoids^14,15^, we described gingerols with one aromatic ring gingeroids. Among them, the concentration of 6-gingerol was higher than that of other gingerol analogs in ginger rhizomes and showed a decreasing tendency from the Rh1 to Rh5 stages (Fig. 4C). By using triple quadrupole mass spectrometry, we were able to quantify gingeroids and curcuminoids in the ginger rhizome samples. We found that 6-gingerol and tetrahydrocurcumin had the highest concentrations in Rh1 (1082.4 ± 413.2 μg/g, 26.7 ± 4.3 μg/g) and the lowest concentrations in Rh5 (433.0 ± 107.3 μg/g, 8.5 ± 2.3 μg/g) (Supplementary Fig. S23).

Based on the data from our metabolomic analysis and previous literature^6,16–18^, we propose a backbone biosynthetic pathway for gingerol analogs (Fig. 4C). We suspect that phenylalanine is catalyzed to form feruloyl-CoA through a network far more complex than previously reported. This network is shared by the gingerol and monolignol biosynthetic pathways^19^, and all the enzyme-encoding genes in this pathway from eight families, *PAL*, *C4H*, *4* *CL*, *CST*, *C3’H*, *C3OMT*, *CCOMT*, and *CSE*, were found in the ginger genome (Supplementary Table S27). Feruloyl-CoA is subsequently converted into various gingeroids and curcuminoids. Based on the structural similarity of these gingeroids and curcuminoids, we propose a generation order for these metabolites and their corresponding enzyme-encoding genes, including *PKS*, *AOR*, *DHN*, and *DHT* (Supplementary Table S27). *PKSs* have been proposed to catalyze the formation of curcumin in previous studies^17^ and are proposed to catalyze the formation of 6-dehydrogingerdione here. *AORs* have only been reported in bacteria^20,21^ and were identified herein ginger by BLAST analysis. *DHNs* and *DHTs* are hypothetical enzyme-encoding genes proposed in this study.

### Key factors for gingerol biosynthesis

**Fig. 5:**
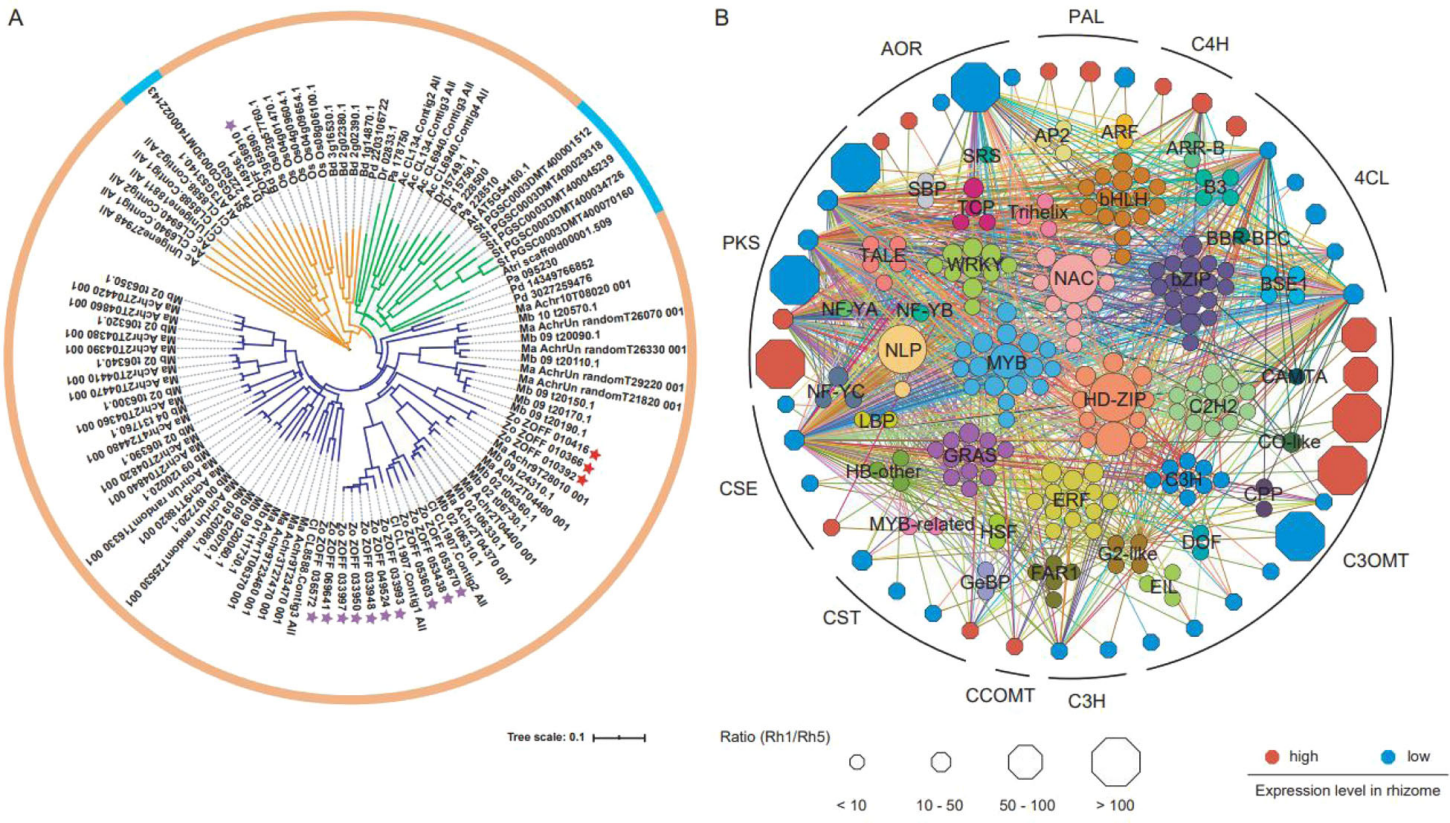
Interaction of key genes in gingerol biosynthesis. **A** Phylogeny of C3OMT genes among 13 plant species, including *Brachypodium distachyon*, *Dioscorea rotundata*, *Phalaenopsis aphrodite*, *Phoenix dactylifera*, *Acorus calamus*, *Curcuma longa*, *Arabidopsis thaliana*, *Solanum tuberosum*, *M. acuminate*, *M. balbisiana*, *O. sativa*, *Z. officinale*, and *A. trichopoda*. Genes from monocots and dicots are denoted by different colored circles. The C3OMT genes were grouped into 3 clades/subfamilies, each of which is shown in a different color. The C3OMT genes in ginger are marked with an asterisk. The unique C3OMT genes in ginger are indicated by red asterisks. **B** Coexpression network connecting structural genes in gingerol biosynthesis with transcription factors. The color-filled hexagons represent the structural genes associated with gingerol biosynthesis that were highly (red) or lowly (blue) expressed in ginger rhizomes. The size of the hexagon represents the FPKM value ratio of each gene between Rh1 and Rh5. Expression correlations between TFs (colored solid circles) and gingerol-related genes (colored solid hexagons) are shown with colored lines (Pearson’s correlation test, *P* < 0.05)

Transcriptome analysis was performed to identify differentially expressed genes (DEGs) in ginger rhizomes at five developmental stages, as well as in the roots and stems. A total of 6690 genes were significantly downregulated in ginger rhizomes from Rh1 to Rh5, whereas 773 genes were upregulated from Rh1 to Rh5 (Supplementary Table S28–30). The DEGs of the five developmental stages of rhizomes were organized into 8 modules according to weighted gene coexpression network analysis (WGCNA) (Supplementary Fig. S24). Transcriptome and metabolite correlation analysis showed that the tissue expression patterns of these 10 gene families were highly correlated with the accumulation of gingerols and curcuminoids (Fig. 4C, Supplementary Fig. S25, Supplementary Table S27). Numerous transcription factors (TFs), including DOF, CPP, NLP, bZIP, C3H, and MYB TFs, showed similar expression patterns as these gene family members (Fig. 5B and Supplementary Table S31).

It has been reported that feruloyl-CoA, the direct precursor of gingeroids and curcuminoids, can be synthesized from caffeic acid through two branch pathways (Fig. 4C)^18^. Interestingly, *4CL*s are present in both branches, whereas *C3OMTs* and *CCOMTs* function in separate branches. Notably, *C3OMTs* exhibited predominant expression in old rhizomes, whereas *CCOMTs* showed no obvious difference in expression levels between old rhizomes and new rhizomes. In addition, phylogenetic analysis showed that three ginger *C3OMT* genes (*C3OMT2*, *3*, and *13*) formed a unique clade (Fig. 5A). Thus, we speculated that *C3OMT*s may play an important role in feruloyl-CoA biosynthesis and that feruloyl-CoA is synthesized mainly through the following subpathway in the ginger rhizomes: caffeic acid→ferulic acid→feruloyl CoA.

We inspected the genome dataset to confirm the copy number and chromosome locations of genes involved in the gingeroid biosynthesis pathway (Supplementary Tables S27 and S32). We found that the *AOR* and *PKS* gene families were significantly expanded in ginger and exhibited more tandem repeats on chromosomes (Supplementary Figs. 26–28 and Supplementary Table S18). In addition, phylogenetic analysis uncovered the genetic relationships between ginger *PKS*s and their orthologs in 13 other plant species. *PKS*s from the four Zingiberaceae species clustered into two groups, *DCS/CURS* and *CHS* (Supplementary Fig. S25). It has been reported that DCS/CURS can catalyze the production of curcumin from feruloyl-CoA, whereas CHS can catalyze the production of chalcone from coumaroyl-CoA. Similar to curcuminoids, gingeroids are also synthesized from feruloyl-CoA, and we, therefore, speculated that some *DCS/CURS*s in the ginger genome may be responsible for the synthesis of gingeroids, while others are responsible for the synthesis of curcuminoids.

## Discussion

Compared with animal genomes, plant genomes are more complex because of their high heterozygosity and high ploidy caused by distant hybridization and self-incompatibility^22^. Furthermore, plant genomes are relatively large, making them more difficult to assemble. Nonetheless, researchers have made various attempts to assemble autopolyploid genomes in animals and plants. It is well known that only one set of chromosomes from diploid species can be assembled^23^. For instance, the genome of the hexaploid sweet potato *Ipomoea batatas* was assembled with a specifically developed algorithm based on ~296 Gb of paired-end next-generation sequencing reads with approximately 67× coverage^24^. In addition, allele-defined chromosome-level genomes of autotetraploid cultivated alfalfa *Medicago sativa* and haploid (1n = 4x = 32) sugarcane *S. spontaneum* were assembled using PacBio long reads and a Hi-C-based physical map^25,26^. In our study, a combined sequencing strategy (PacBio CLR, Illumina short reads, and Hi-C) was used to generate a haplotype-resolved reference genome assembly for diploid ginger. Successful assembly of the haplotype-resolved diploid genome may be due to its high heterozygosity. The heterozygosity of ginger is higher than that of previously reported plant genomes, even that of the tea plant (*Camellia sinensis*) (2.8%)^27,28^. The high level of variation in the ginger genome is comparable to that in bananas, and this is also helpful for haplotype phasing^19^. Two types of technical workflows were used for the assembly of the abovementioned genomes: (1) defining phasing based on SNPs (similar to the sweet potato genome) or (2) direct assembly of long reads (Hi-C-assisted allelic assembly, 10× phasing assembly, etc.) (similar to the sugarcane genome)^23^. In our case, high-coverage long reads and Hi-C mapping may have been the critical factors for haplotype phasing. In another study, a phased diploid reference genome for *Vanilla planifolia* ‘Daphna’ (Daphna) was assembled de novo from a combination of Oxford Nanopore Technologies (ONT) long reads, Illumina short reads, and Hi-C chromatin data^29^. Notably, the expression levels of these allelic genes in our analyses did not differ significantly between the two haplotypes, while most of the differentially expressed loci were mainly enriched in metabolic pathways.

In this study, a relatively complete biosynthetic pathway for gingeroids and curcuminoids was constructed based on synergistic analysis of multiple data types, including ginger genome sequencing, transcriptomics, and metabolomics. This complicated pathway consisted of two parts: the upstream part from L-phenylalanine to feruloyl-CoA and the downstream part from feruloyl-CoA to gingeroids and curcuminoids. Notably, the upstream pathway is shared with monolignol biosynthesis, and the monolignol biosynthesis pathway has been clearly demonstrated and is highly conserved in all vascular plants. Enzymes involved in the upstream pathway are relatively conserved in most plants^30^. However, the downstream pathway is involved in the synthesis of dozens of specific compounds in ginger and/or turmeric that are known as gingeroids and curcuminoids^7^. Therefore, the enzymes in the downstream pathway may be unique to Zingiberaceae plants. For example, PKS and AOR are the most important enzymes in the downstream pathway, catalyzing the formation of gingeroids and/or curcuminoids from feruloyl-CoA^17^. Based on the phylogenetic analysis, we suggest that a specific *PKS* subgroup (which we have called *DCS/CURS*) exists in Zingiberaceae plants and plays a critical role in the synthesis of gingeroids and curcuminoids. Previous reports have described a two-step reaction in which feruloyl diketide-CoA is produced from feruloyl-CoA by DCS, and gingeroids and curcuminoids are catalyzed from feruloyl diketide-CoA by CURS^17^. Among seventeen *PKS*s in the ginger genome, five (*PKS-6*, *-7*, *-10*, *-15*, and *-16*) were clustered into one branch with the turmeric *DCS*, and five (*PKS-1*, *-2*, *-4*, *-8*, and *-14*) were clustered into another branch with the turmeric *CURS*. The remaining 7 ginger *PKS*s were clustered with *CHS*. Our results, therefore, suggest that the 10 ginger *DCS/CURS* complexes (*PKS-1*, *-2*, *-4*, *-6*, *-7*, *-8*, *-10*, *-14 -15*, and *-16*) are responsible for the synthesis of gingeroids and curcuminoids. AOR exhibits NADPH-dependent reductase activity and is also called curcumin reductase (CurA); it can catalyze the conversion of curcumin to tetrahydrocurcumin in bacteria^20,21^ and has potential curcumin reductase activity in plants. Compared with curcuminoids, gingeroids share similar molecular structures. We, therefore, speculated that the synthesis of gingeroids and curcuminoids may be regulated by orthologous *PKS* and *AOR* genes in both ginger and turmeric, consistent with previous studies on gingeroid biosynthesis^16^. Taken together, our results suggest that the specific evolution of the *PKS* gene family may have conferred a novel function to Zingiberaceae plants beyond the synthesis of chalcones, which are also involved in the synthesis of gingeroids and curcuminoids.

Transcription factors play a critical role in regulating gene expression. Based on our comprehensive genome, transcriptome, and metabolome data in ginger, many types of TFs were found to be involved in the regulation of key gingeroids, including some of those that encode DOF, CPP, NLP, bZIP, C3H, and MYB (Fig. 5B and Supplementary Table S32). It is well known that these TFs function as regulators in the plant phenolic biosynthesis pathway^31^. The expression pattern of bHLHs was also found to be closely related to the expression patterns of PKSs and AORs, which form ternary complexes with WD40 and MYB activators or repressors that regulate flavonoid biosynthesis^32^. Several TFs that are responsible for biotic and/or abiotic stress, such as HD-ZIP, WRKY, C2H2, C3H, NAC, and ERF family members, also showed a strong association with the expression of gingeroid biosynthesis genes, in agreement with previous reports showing the anti-insect and antimicrobial activities of these natural products^33,34^.

Our results also showed that the *C3OMT* gene family evolved into a specific subgroup during ginger genome evolution and that *C3OMTs* were preferentially expressed in mature rhizomes. Notably, two gene families involved in the biosynthesis of gingerol analogs (*PKS* and *AOR*) were significantly expanded in the ginger genome. These genes exhibited a tandem repeat distribution on ginger chromosomes and showed higher expression levels in old rhizomes. Correlation analyses of transcriptomic and metabolomic data revealed correlations between specific gingerol biosynthetic gene family members. Thus, the expansion, mutation, and tissue-specific expression patterns of *PKS*, *AOR*, and *C3OMT* are responsible for the specific synthesis of gingeroids in ginger.

## Materials and methods

### Plant materials and genome sequencing

Seedlings of ginger *Z. officinale* ‘Zhugen’ were grown in the greenhouse of the Institute of Special Plants, Chongqing University of Arts and Sciences (29°14’ N, 105°52’ E) beginning in April 2018. The growth conditions were 25 ± 3 °C, relative humidity 60 ± 5%, and 14 h light (220 ± 10 μEm^−2^ s^−1^). Young leaf samples were collected in July 2018, and high-molecular-weight (HMW) DNA for genome sequencing was extracted using the DNAsecure Plant Kit (TIANGEN). Three short insert libraries (one 270 bp and two 500 bp) were constructed following the manufacturer’s instructions (Illumina, San Diego, CA) and sequenced in 150-bp paired-end mode on the Illumina HiSeq X-Ten platform. For single-molecule real-time (SMRT) long-read sequencing, five 20-kb insert libraries were constructed, and a total of 29 SMRT cells with 285.81 Gb of sequence data (167-fold coverage of the genome) were sequenced on the PacBio Sequel platform. The mean length and N50 length of the subreads were 12.5 kb and 19.9 kb, respectively. The Hi-C library was generated according to a published protocol^35^. In brief, 2 g of young leaves was cross-linked in situ in 1% formaldehyde solution. Chromatin was extracted and digested with *MboI* (New England Biolabs), and the DNA ends were labeled, biotinylated, diluted, and randomly ligated. The DNA fragments were enriched and quality-checked to ensure that they were suitable for library preparation. Finally, three sequencing libraries were constructed and sequenced on the BGISEQ-500 platform in 100-bp paired-end mode. For RNA sequencing and metabolomic analysis, plants were randomly selected after 180 days of growth. Rhizomes were collected at five developmental stages (Rh1–Rh5) based on their growth segments. Aboveground parts (St, aerial stem and leaves) and roots (Ro) were also collected. Three biological replicates of each tissue were collected, and each replicate consisted of pooled samples from five different plants. All samples were rinsed with Milli-Q water and immediately stored in liquid nitrogen.

### K-mer analysis and genome assembly

To determine the genome characteristics of ginger, K-mer analysis was performed using jellyfish^36^ and Genomescope 1.0^37^. FALCON is a hierarchical, haplotype-aware genome assembly tool. Falcon (v0.3.0)^38^ was then used to assemble the initial contigs using default parameters with several exceptions: “-t 20 -h 300 -e.75 -w 8 -l 2000 -s 1000 -k 17” for read correction and “-v -D 24 -M 32 -h 1050 -e.94 -l 3000 -s 1000 -k 25 -B 4.” The initial contigs from all sequenced PacBio long reads were polished with Quiver^39^ using the Arrow algorithm (https://github.com/PacificBiosciences/GenomicConsensus). Illumina short reads were then aligned to the corrected PacBio contigs using BWA-MEM^40^, and Pilon (v1.22)^41^ was used to correct errors in the contigs. The Hi-C sequencing data were mapped onto the assembled contigs by Juicer (v1.5)^42^ and 3D-DNA^43^ to anchor contigs onto chromosomes by default parameters. The quality and completeness of the assembled genome were evaluated by Benchmarking Universal Single-Copy Orthologs (BUSCOs v3, embryophyta_odb10)^44^ and the LTR assembly Index (LAI v beta 3.2)^45^.

### Haplotype comparison

SNP calling was performed to evaluate sequence variations between haplotype 0 and haplotype 1^24^. The corrected PacBio reads from haplotype 0 and haplotype 1 were aligned using blasr (https://github.com/mchaisso/blasr). The number of matched SNPs and mismatched SNPs on each read were counted. The Ka/Ks ratio was calculated using *M. acuminata* orthologs as the outgroup. MUSCLE (v3.8.31) was used to construct multiple nucleotide sequence alignments from the CDSs of the orthologous gene sets. Ka/Ks ratios of codons were calculated using Codeml in the PAML package. To identify the allelic genes between the two haplotypes, we applied the MCScan package to construct syntenic blocks based on well-aligned genes. First, an all-vs-all BLASTP was conducted to align proteins of the two gene sets with the e-value parameters “1e-7”. Then, the proteins were subjected to alignments via MCScan to identify syntenic blocks with the parameters -a -e 1e-5 -u 1 -s 5. Finally, the allelic genes were screened to confirm paired regions on homologous haplotypes. PacBio read coverage for each chromosome of the two phases was obtained by BamTools and visualized using the R package. MUMmer was used to determine the most accurate position for inversions. The coverage of PacBio reads around the reversed regions, especially the breakpoints, was accessed by BWA and visualized by IGV. The expression of homoeologous genes from the two phases was calculated as fragments per kilobase of transcript per million mapped reads (FPKM). Genes with FPKM values >0.5 for all samples were taken as expressed genes. The phase 0/phase 1 expression ratio was calculated and log-transformed as log_10_((FPKM haplotype0)/(FPKM haplotype1)). The differential expression of alleles was calculated using Noiseq^46^ with a threshold of ‘probability ≥ 0.8 and relative change ≥ 2’. GeneWise (2.4.1) was used to identify pseudogenes and fragmented genes in each phase.

### Gene annotation and analysis of repetitive sequences

The assembled contig sequences before phasing (3,089,604,979 bp) were used for homology-based, de novo, and transcriptome-based gene predictions. First, the homologous proteins from *M. acuminata*, *S. bicolor*, and *O. sativa* were used to identify proteins in the repeat-masked ginger genome reference sequence with Exonerate^47^ (v2.2.0, parameters: --model protein2genome -percent 50 -minintron 10, -maxintron 50000, -align_rate 0.25, -bestn 10). Second, Augustus (v3.2.1)^48^ was used to train a coding gene model for de novo predictions. Third, the ginger transcriptome unigenes assembled by Trinity were mapped to the ginger genome with Exonerate. Finally, all gene prediction data were combined into a consensus gene set using the MAKER pipeline (v3.31.8)^49^.

To infer the insertion time of LTR retrotransposons, full-length LTR retrotransposons from six species (*Z. mays, O. sativa, S. bicolor, Z. officinale, M. acuminata*, and *M. balbisiana*) were identified using LTRharvest and LTRdigest incorporated into Genome Tools (v1.5.8)^50^. The timing of insertion was analyzed based on the divergence of the 5′ and 3′ LTR sequences of each copy. The 5′ and 3′ LTRs were aligned using MUSCLE (v3.8.31)^51^, and the substitutions per nucleotide site were calculated. The insertion time was estimated with an average base substitution rate of 6.5e^−9^ Ks/year^52^.

### Gene family identification and phylogenetic analysis

A total of 1,112 single-copy orthologous genes were identified between *Z. officinale* and nine published plant species (*A. trichopoda*, *A. comosus*, *A. officinalis, C. nucifera*, *L. chinense*, *M. acuminata*, *M. balbisiana*, *O. sativa*, and *S. bicolor*) using OrthoMCL (v2.0.9)^53^. Each of the gene sets from the 10 species was filtered using two conditions. First, if there were multiple alternatively spliced transcripts in a gene, only the longest transcript was retained. Second, genes encoding proteins less than 50 amino acids in length were excluded. The similarity of protein sequences was assessed by all-versus-all BLASTP (v2.2.26)^54^ with an E-value <1e^−5^.

Using extracted single-copy orthologs from the gene clustering analysis, multiple alignments of protein sequences were constructed in MAFFT (v7.0) with default parameters^55^. We performed multiple alignments of protein sequences for each gene family with MUSCLE and converted the protein alignments to CDS alignments using a Perl script. We extracted phase 1 sites of all single-copy orthologous genes in each species and concatenated them to one supergene for phylogenetic construction. We constructed a phylogenetic tree using PhyML^56^. Finally, TreeBest (https://github.com/Ensembl/treebest) was used to define the root with *A. trichopoda* as the outgroup. Divergence times among these species were calculated using MCMCTREE in the PAML package (v4.5)^57^. Three calibration points for the divergence analysis were obtained from the TimeTree database (http://www.timetree.org/). The expansion and contraction of gene families were calculated with CAFE (v3.1)^58^.

### Identification of WGD in ginger

To obtain syntenic blocks, protein sequences from ginger, grape, and *M. acuminata* were compared using BLASTP (v2.2.26) with an E-value <1e^−5^. The collinearity of more than five genes was defined as a syntenic block in MCScanX (v0.8)^59^. The 4dTv (fourfold degenerate synonymous sites of the third codon) of syntenic segments was calculated from the concatenated alignments. The distribution of the 4dTv values was plotted, and the peak was used to infer the WGD. To identify WGD, Ks-based distributions of all paralogous genes in the ginger and *M. acuminata* genomes were constructed. MUSCLE (v3.8.31) was used to align each gene family, and the CODEML program in the PAML package (v4.5) was used to estimate Ks for all pairwise comparisons within a gene family.

### Transcriptomic analysis and key factor identification for gingerol biosynthesis

In brief, oligo(dT)-attached magnetic beads were used to purify total mRNA. Purified mRNA was fragmented into small pieces in fragmentation buffer at an appropriate temperature. First-strand cDNA was generated using random hexamer-primed reverse transcription, followed by second-strand cDNA synthesis. A-Tailing Mix and RNA Index Adapters were added. The cDNA fragments were amplified by PCR, and products were purified with Ampure XP Beads and then dissolved in elution buffer (EB) solution. For quality control, the PCR product was validated on an Agilent Technologies 2100 bioanalyzer. The double-stranded PCR products from the previous step were denatured and circularized by the splint oligo sequence to obtain the final library. The final single-strand circularized DNA (ssCirDNA) library was amplified with phi29 to make DNA nanoballs (DNBs), which had more than 300 copies of one molecule. Finally, DNBs were loaded into the patterned nanoarray, and 150-bp paired-end reads were generated on the DNBseq platform. Gene coexpression network analysis was performed with WGCNA^60^. Correlations between transcriptome and metabolome data were calculated following the method of Song et al.^61^.

Based on previous studies^16–21^, all the enzyme-encoding genes involved in curcumin biosynthesis and the network shared by the gingerol and monolignol biosynthetic pathways were retrieved from the NCBI and UniProt databases. To identify homologs of these genes in ginger, a BLAST search (BLASTP) was carried out against the ginger genome with an e-value cutoff of 1e−5, alignment coverage ≥50%, and identity >50%. The relative transcription factors were identified via a BLASTP search from PlantTFDB (http://planttfdb.gao-lab.org/tf.php?sp=Ppe&did=Prupe. I00450 0.1.p) using gene sequences from WGCNA modules as queries.

### UHPLC/UPLC-MS/MS analysis of the ginger extract

Dried ginger samples (0.3 g each, 8 biological replicates) were pulverized with a tissue grinder. The resulting powder (0.5 g) was added to 80% methanol solution (2 ml) and homogenized for 2 h, followed by ultrasonic extraction at 100 kHz for 90 min. After centrifugation at 14,000 × *g* for 20 min, the supernatant was filtered through a 0.22-μm membrane.

For UHPLC-MS/MS analysis, samples were loaded into a Q Exactive Focus mass spectrometer (Thermo Scientific, USA) with a Hypersil GOLD aQ column (100 × 2.1 mm, 1.9 μm). The mobile phase consisted of 0.1% formic acid/acetonitrile solution (v/v, solvent A) and a 0.1% formic acid aqueous solution (v/v, solvent B). The flow rate was 0.4 mL/min, and the injection volume was 2 μL. A linear gradient with the following proportions of phase A (time in min, A%) was used: (0, 5), (2, 5), (25, 95), (28, 98), (28.1, 5), and (30, 5). Mass spectra were acquired in positive and negative ionization modes through full MS and higher-energy collisional dissociation (HCD) data‐dependent MS/MS analysis (full MS‐ddMS2). The mass range was from *m*/*z* 100 to 1500. The resolution was set to 70,000 (FWHM at *m*/*z* 200) for the full MS scans and to 17,500 (FWHM at *m*/*z* 200) for HCD MS/MS scans. The normalized collision energy (NCE) was set from 15% to 60%. The spray voltage, vaporizer temperature, capillary temperature, sheath gas flow rate, and auxiliary gas flow rate were 3.5/3.2 kV (+/−), 300 °C, 350 °C, 45 arbitrary units, and 15 arbitrary units, respectively. All datasets from the Q Exactive analysis were processed with Compound Discoverer 3.0 software (Thermo Scientific, USA). The following compounds were eliminated: compounds without name annotations, compounds with group CV values above 40, compounds without secondary spectra, and compounds with a chemspider value and mzcloud value of 0.

For UPLC-MS/MS analysis, samples were loaded into the Xevo TQ-S micro Triple Quadrupole Mass Spectrometer (Waters, USA) with a C18 column (Acquity BEH, 50 × 2.1 mm, 1.7 µm). The mobile phases were acetonitrile solution (solvent A) and 0.1% formic acid aqueous solution (solvent B). The flow rate was 0.2 mL/min. A linear gradient with the following proportions of phase A (time in min, A%) was used: (0, 10), (6, 90), (8, 90), (8.5, 10), and (10, 10). The following conditions were used for the electrospray ionization (ESI) source: capillary voltage 3.0 kV, cone voltage 25 V, source temperature 150 °C, desolvation temperature 350 °C, and nebulizer gas 650 L/h N_2_. The collision energies were optimized and ranged from 10 to 40 eV for individual analytes. The ESI source was operated in positive ion mode. Instrument control and data processing were performed using MassLynx software (version 4.1, Waters, USA). Standard solutions of 6-gingerol and tetrahydrocurcumin (Yuanye Bio-Technology, Shanghai, China) were prepared at concentrations ranging from 0.1 μg/mL to 10 μg/mL and 1 ng/mL to 100 ng/mL in methanol, respectively. Analyte identity was determined based on retention time and mass spectra, and quantification was based on the analyte to standard area ratios.

## Supplementary information

supplymentary tables

Supplementary Fig. S1

Supplementary Fig. S2

Supplementary Fig. S3

Supplementary Fig. S4

Supplementary Fig. S5

Supplementary Fig. S6

Supplementary Fig. S7

Supplementary Fig. S8

Supplementary Fig. S9

Supplementary Fig. S10

Supplementary Fig. S11

Supplementary Fig. S12

Supplementary Fig. S13

Supplementary Fig. S14

Supplementary Fig. S15

Supplementary Fig. S16

Supplementary Fig. S17

Supplementary Fig. S18

Supplementary Fig. S19

Supplementary Fig. S20

Supplementary Fig. S21

Supplementary Fig. S22

Supplementary Fig. S23

Supplementary Fig. S24

Supplementary Fig. S25

Supplementary Fig. S26

Supplementary Fig. S27

Supplementary Fig. S28
