# Correction: Haplotype-resolved genome of diploid ginger (*Zingiber officinale*) and its unique gingerol biosynthetic pathway

**DOI:** 10.1038/s41438-021-00700-1

**Published:** 2021-10-28

**Authors:** Hong-Lei Li, Lin Wu, Zhaoming Dong, Yusong Jiang, Sanjie Jiang, Haitao Xing, Qiang Li, Guocheng Liu, Shuming Tian, Zhangyan Wu, Bin Wu, Zhexin Li, Ping Zhao, Yan Zhang, Jianmin Tang, Jiabao Xu, Ke Huang, Xia Liu, Wenlin Zhang, Qinhong Liao, Yun Ren, Xinzheng Huang, Qingzhi Li, Chengyong Li, Yi Wang, Baskaran Xavier-Ravi, Honghai Li, Yang Liu, Tao Wan, Qinhu Liu, Yong Zou, Jianbo Jian, Qingyou Xia, Yiqing Liu

**Affiliations:** 1 College of Landscape Architecture and Life Science/Institute of Special Plants, Chongqing University of Arts and Sciences, Yongchuan, Chongqing China; 2 Engineering Research Center for Special Plant Seedlings of Chongqing, Chongqing University of Arts and Sciences, Yongchuan, Chongqing China; 3 State Key Laboratory of Silkworm Genome Biology, Biological Science Research Center, Southwest University, Beibei, Chongqing China; 4 BGI Genomics, BGI-Shenzhen, Shenzhen, Guangdong China; 5 College of Biology and Food Engineering, Chongqign Three Gorges University, Wanzhou, Chongqing China; 6 Department of Entomology and MOAKey Lab of Pest Monitoring and Green Management, College of Plant Protection, China Agricultural University, Haidian, Beijing China; 7 Jinan Second Agricultural Science Research Institute, Jinan, Shandong China; 8 Savari Research Foundation, Mela Ilandai Kulam, Tamil Nadu India; 9 Institute of Advanced Interdisciplinary Studies, Chongqing University, Chongqing, China; 10 Fairy Lake Botanical Garden and Chinese Academy of Sciences, Shenzhen, Guangdong China; 11 Ningyang Science and Technology Bureau, Taian, Shandong China; 12 College of Horticulture and Gardening, Yangtze University, Jingzhou, Hubei China

**Keywords:** Genome, Genomics

Correction to: *Hortic. Res*. 10.1038/s41438-021-00627-7 published online 5 August 2021

After online publication of the article^1^, the authors noticed the affiliation “College of Landscape Architecture and Life Science/Institute of Special Plants, Chongqing University of Arts and Sciences, Yongchuan, Chongqing, China” for author Yiqing Liu was missing.

The original article has been corrected
