# Supplementary material for: Haplotype-resolved genome of diploid ginger (Zingiber officinale) and its unique gingerol biosynthetic pathway
Source: Hortic Res. 2021 Aug 5;8:189. doi: 10.1038/s41438-021-00627-7 (PMC8342499; doi:10.1038/s41438-021-00627-7)
Supplement: Supplementary file 2 — Supplementary Fig. S1 [file 41438_2021_627_MOESM2_ESM.pdf]

a

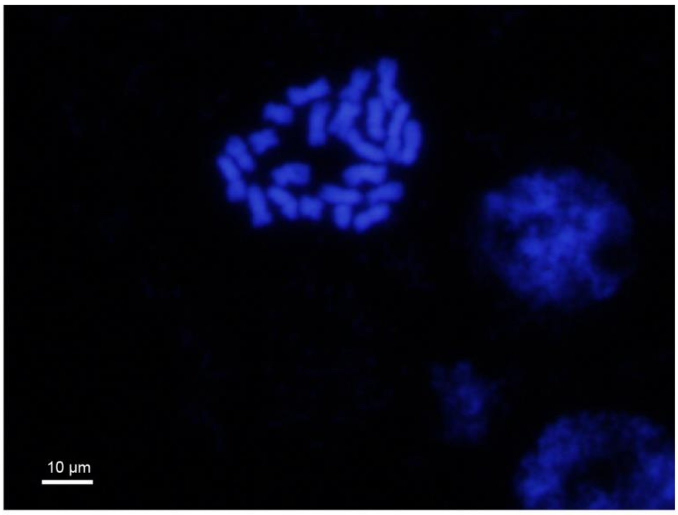

b

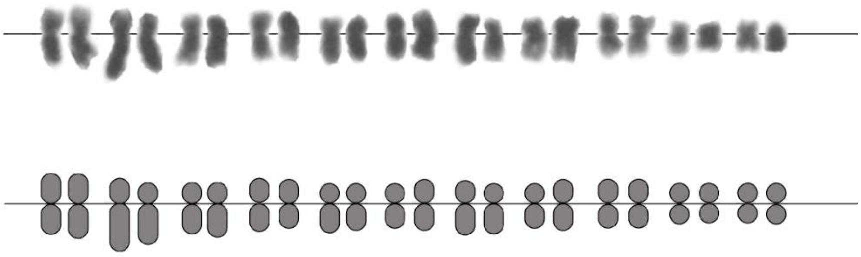

Supplementary Fig. S1 The chromosome numbers and karyomorphology analyses (A) of ginger (*Zingiber officinale*) genomic DNA and (B) the representing schematic diagram.
