# Supplementary material for: Haplotype-resolved genome of diploid ginger (Zingiber officinale) and its unique gingerol biosynthetic pathway
Source: Hortic Res. 2021 Aug 5;8:189. doi: 10.1038/s41438-021-00627-7 (PMC8342499; doi:10.1038/s41438-021-00627-7)
Supplement: Supplementary file 3 — Supplementary Fig. S2 [file 41438_2021_627_MOESM3_ESM.pdf]

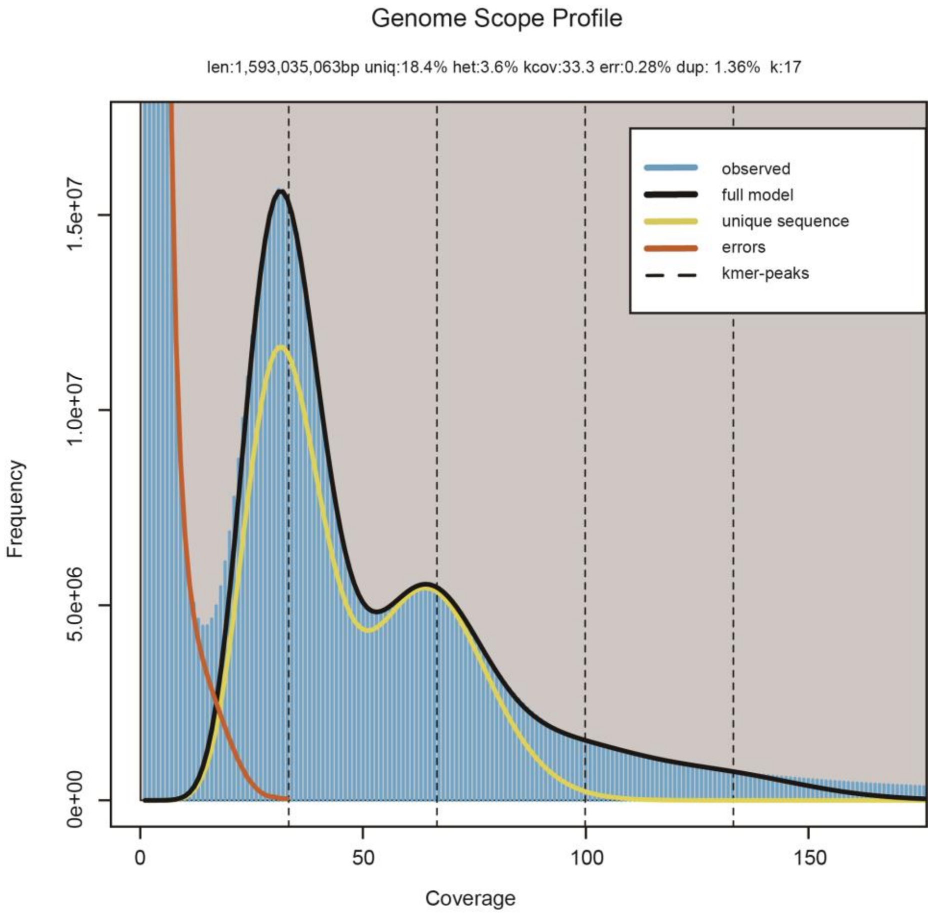

Supplementary Fig. S2 Genome size estimation of ginger by GenomeScope. The ginger genome was estimated to be 1.59 Gb with 3.6% heterozygosity at 17-mer.
