# Supplementary material for: Haplotype-resolved genome of diploid ginger (Zingiber officinale) and its unique gingerol biosynthetic pathway
Source: Hortic Res. 2021 Aug 5;8:189. doi: 10.1038/s41438-021-00627-7 (PMC8342499; doi:10.1038/s41438-021-00627-7)
Supplement: Supplementary file 4 — Supplementary Fig. S3 [file 41438_2021_627_MOESM4_ESM.pdf]

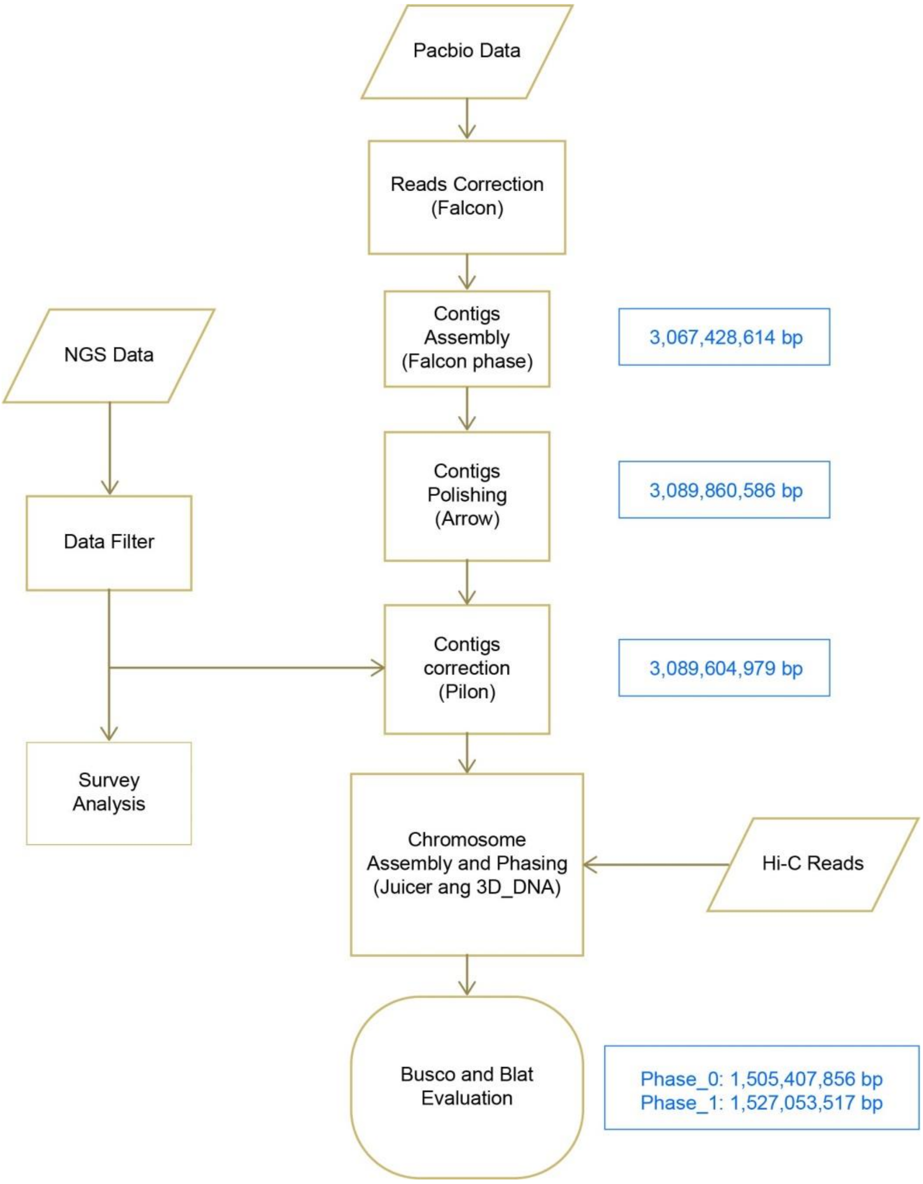

Supplementary Fig. S3 Genome assembly pipeline used in this study. Sequencing data generated from PacBio was assembled and polished. The contigs were corrected by NGS short reads. Hi-C reads were used go generated the chromosome-level contigs and scaffolds.
