# Supplementary material for: Haplotype-resolved genome of diploid ginger (Zingiber officinale) and its unique gingerol biosynthetic pathway
Source: Hortic Res. 2021 Aug 5;8:189. doi: 10.1038/s41438-021-00627-7 (PMC8342499; doi:10.1038/s41438-021-00627-7)
Supplement: Supplementary file 5 — Supplementary Fig. S4 [file 41438_2021_627_MOESM5_ESM.pdf]

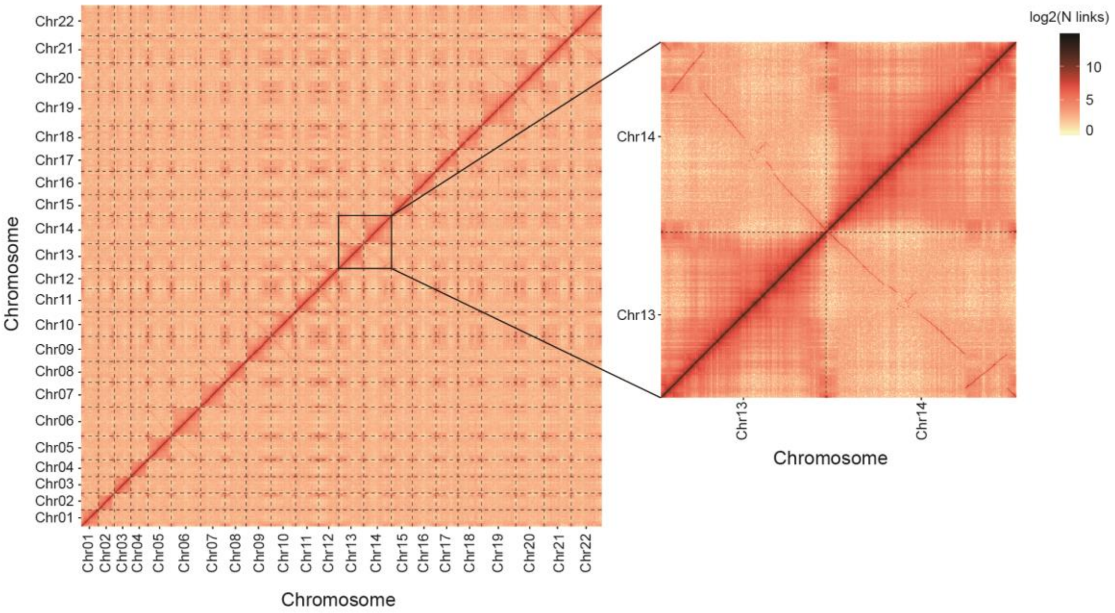

Supplementary Fig. S4 Overview of Hi-C heatmap (A) of the ginger genome showing genome-wide all-by-all interactions. (B) An enlarged graph exhibiting a representative fragmental inversion in chromosomes 13 and 14.
