# Supplementary material for: Haplotype-resolved genome of diploid ginger (Zingiber officinale) and its unique gingerol biosynthetic pathway
Source: Hortic Res. 2021 Aug 5;8:189. doi: 10.1038/s41438-021-00627-7 (PMC8342499; doi:10.1038/s41438-021-00627-7)
Supplement: Supplementary file 6 — Supplementary Fig. S5 [file 41438_2021_627_MOESM6_ESM.pdf]

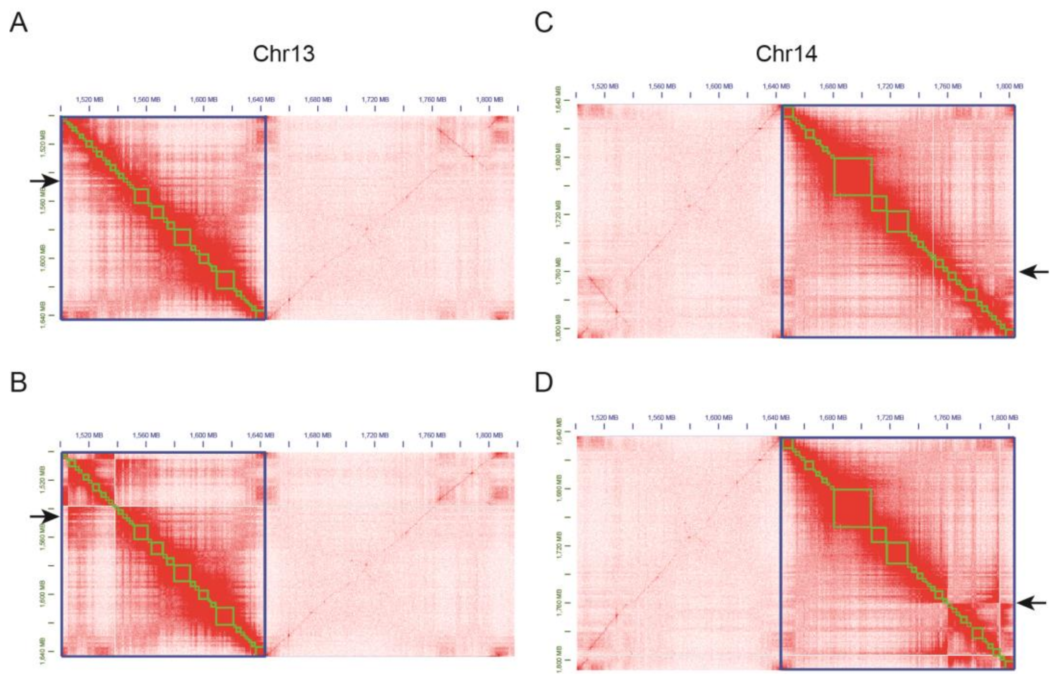

Supplementary Fig. S5 Manual validation of the inversion regions of Chromosome 13 and Chromosome 14. The original enlarged HiC heatmap of Chromosome 13 was shown in Figure A, and the manually reversed graph was shown in Figure B. As indicated by the black arrow, there were more conflictions in the manually reversed version, underlying our assembly was correct and the inversion regions were present in the ginger genome. Similarly, this occurred in Chromosome 14 as indicated by the arrow (Figure C and D).
