# Supplementary figures and images for: Haplotype-resolved genome of diploid ginger (Zingiber officinale) and its unique gingerol biosynthetic pathway
Source: Hortic Res. 2021 Aug 5;8:189. doi: 10.1038/s41438-021-00627-7 (PMC8342499; doi:10.1038/s41438-021-00627-7)

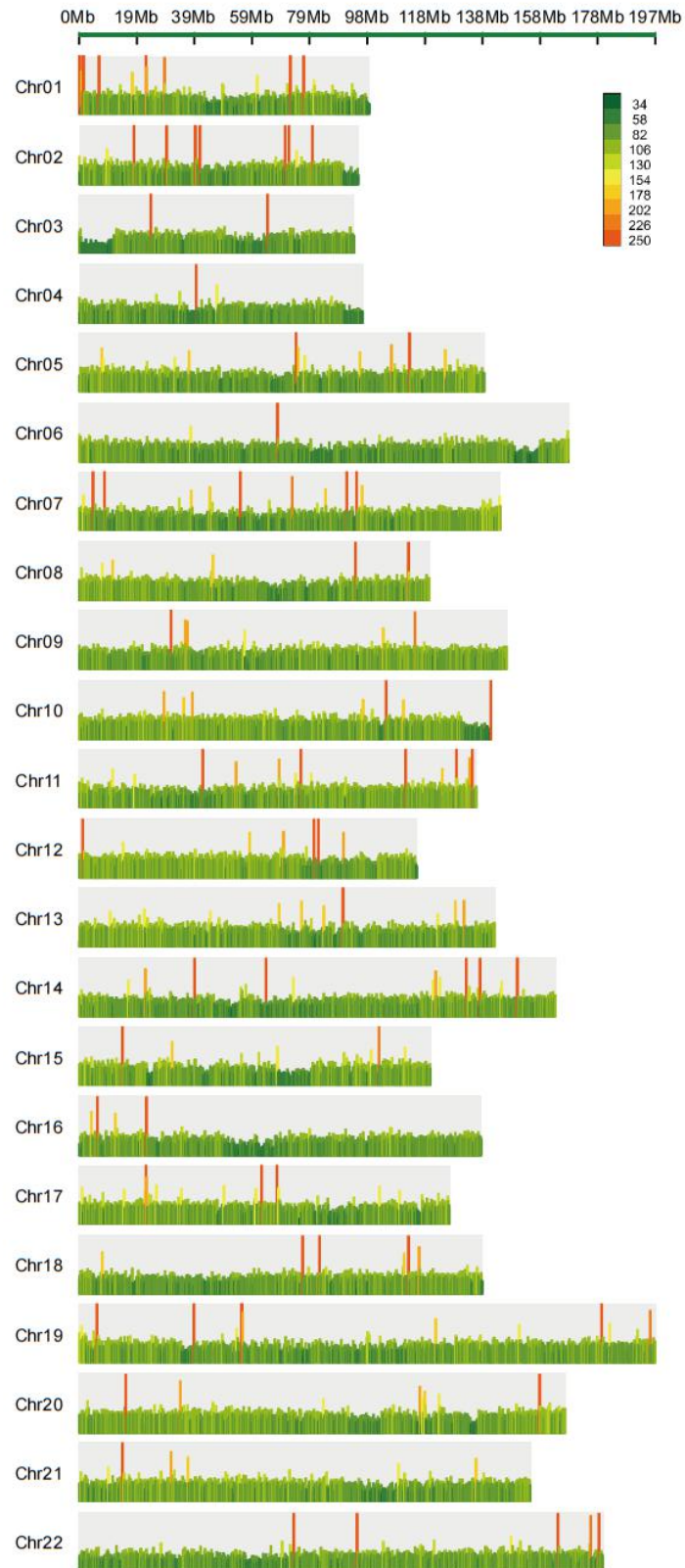

**Supplementary Fig. S6** Reads-coverage (PacBio) of each chromosome of ginger.

Supplement: Supplementary file 7 — Supplementary Fig. S6 [file 41438_2021_627_MOESM7_ESM.pdf]

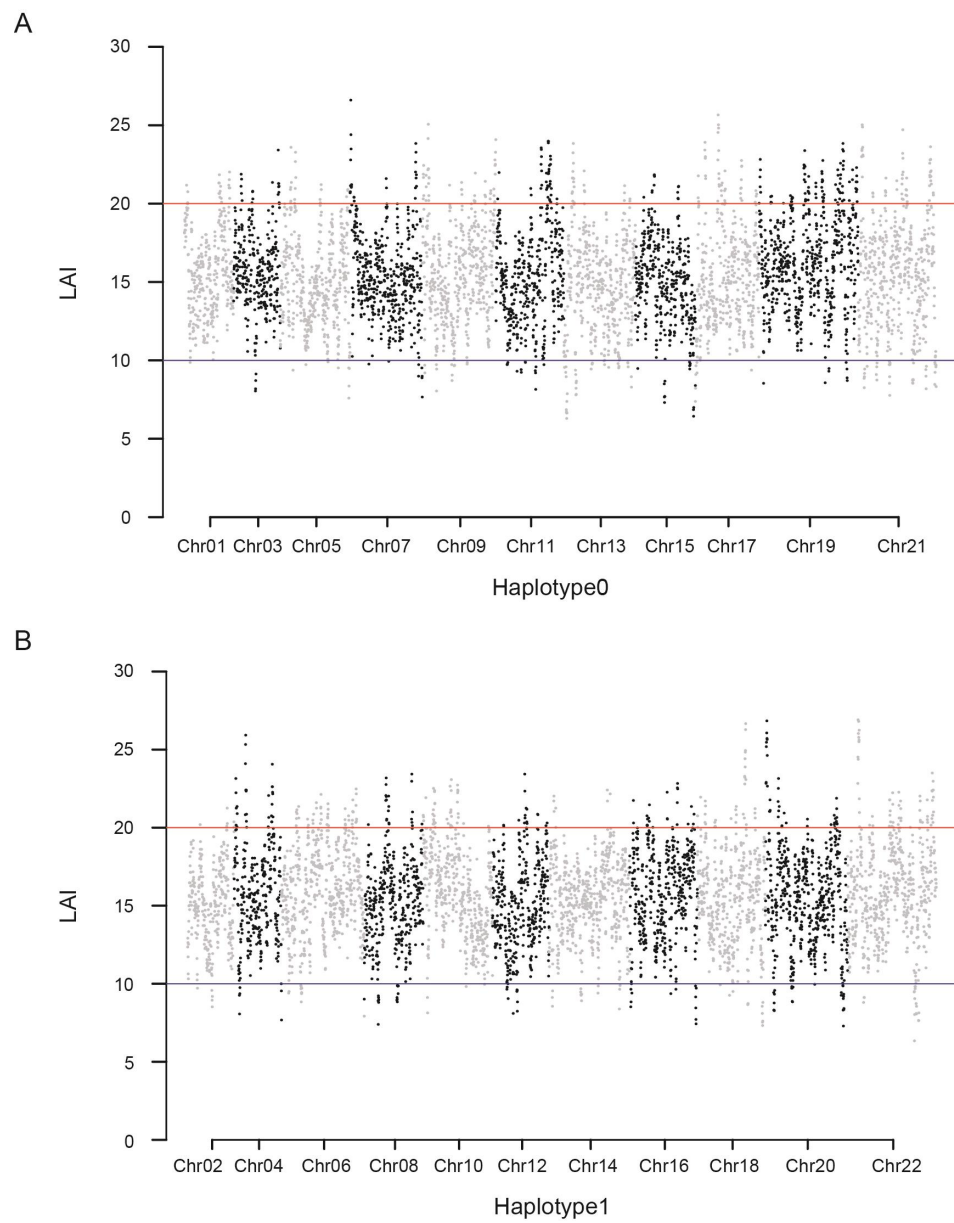

**Supplementary Fig. S8** LAI scores of the two haplotypes.

Supplement: Supplementary file 9 — Supplementary Fig. S8 [file 41438_2021_627_MOESM9_ESM.pdf]
