# Supplementary material for: Haplotype-resolved genome of diploid ginger (Zingiber officinale) and its unique gingerol biosynthetic pathway
Source: Hortic Res. 2021 Aug 5;8:189. doi: 10.1038/s41438-021-00627-7 (PMC8342499; doi:10.1038/s41438-021-00627-7)
Supplement: Supplementary file 8 — Supplementary Fig. S7 [file 41438_2021_627_MOESM8_ESM.pdf]

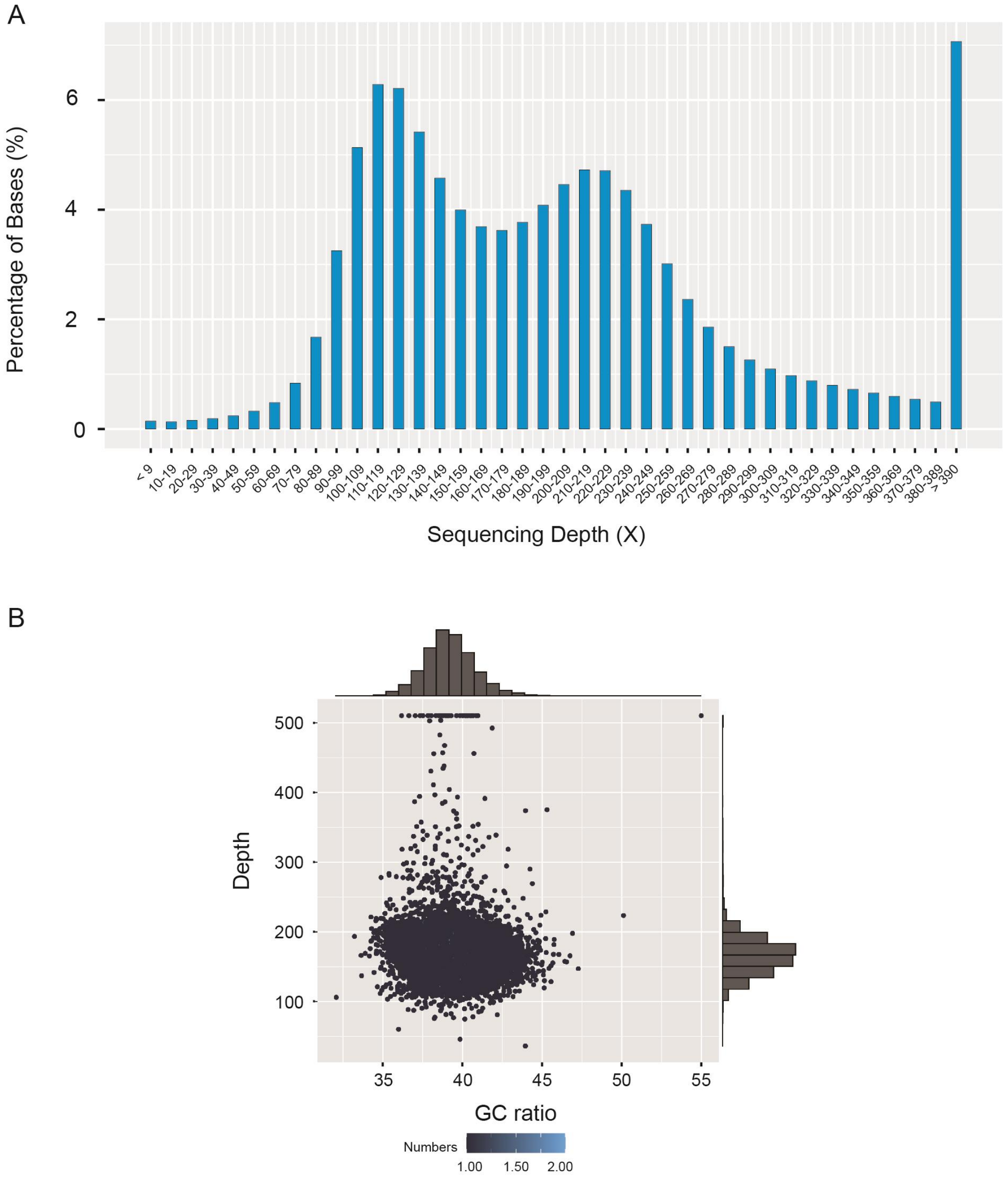

Supplementary Fig. S7 Evaluation of data quality and GC content. (A) The horizontal axis represents sequencing depth of the data and the vertical axis represents percentage of Bases. (B) GC ratio in relation to the sequencing depth.
