# Supplementary material for: Haplotype-resolved genome of diploid ginger (Zingiber officinale) and its unique gingerol biosynthetic pathway
Source: Hortic Res. 2021 Aug 5;8:189. doi: 10.1038/s41438-021-00627-7 (PMC8342499; doi:10.1038/s41438-021-00627-7)
Supplement: Supplementary file 10 — Supplementary Fig. S9 [file 41438_2021_627_MOESM10_ESM.pdf]

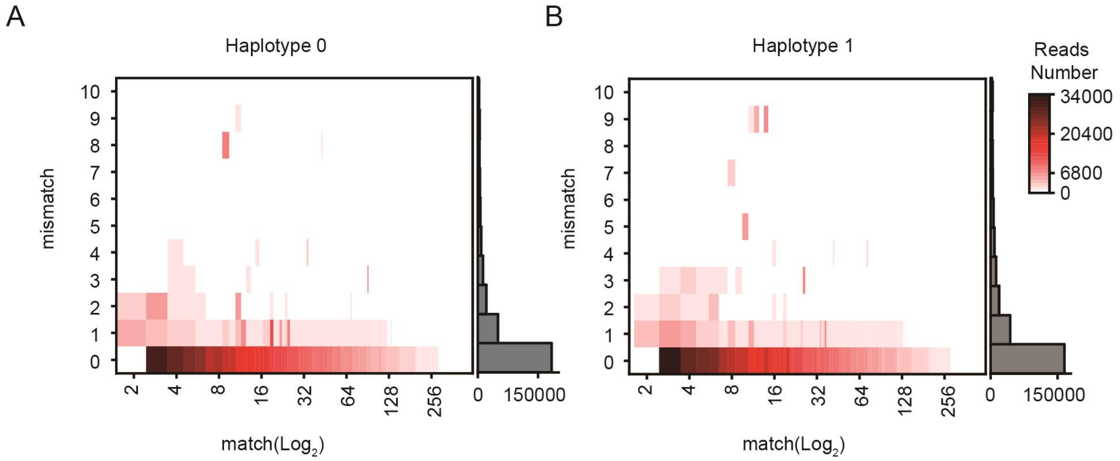

Supplementary Fig. S9 Overlaps between PacBio reads and two haplotype 0 (A) and haplotype 1 (B). The horizontal axis is the number of coinciding polymorphic sites between the haplotype and the PacBio reads. The vertical axis represents the number of varied polymorphic sites in the overlap. Color indicates the frequency of the pairs on an exponential scale, ranging from dark red to light red.
