# Supplementary material for: Haplotype-resolved genome of diploid ginger (Zingiber officinale) and its unique gingerol biosynthetic pathway
Source: Hortic Res. 2021 Aug 5;8:189. doi: 10.1038/s41438-021-00627-7 (PMC8342499; doi:10.1038/s41438-021-00627-7)
Supplement: Supplementary file 15 — Supplementary Fig. S14 [file 41438_2021_627_MOESM15_ESM.pdf]

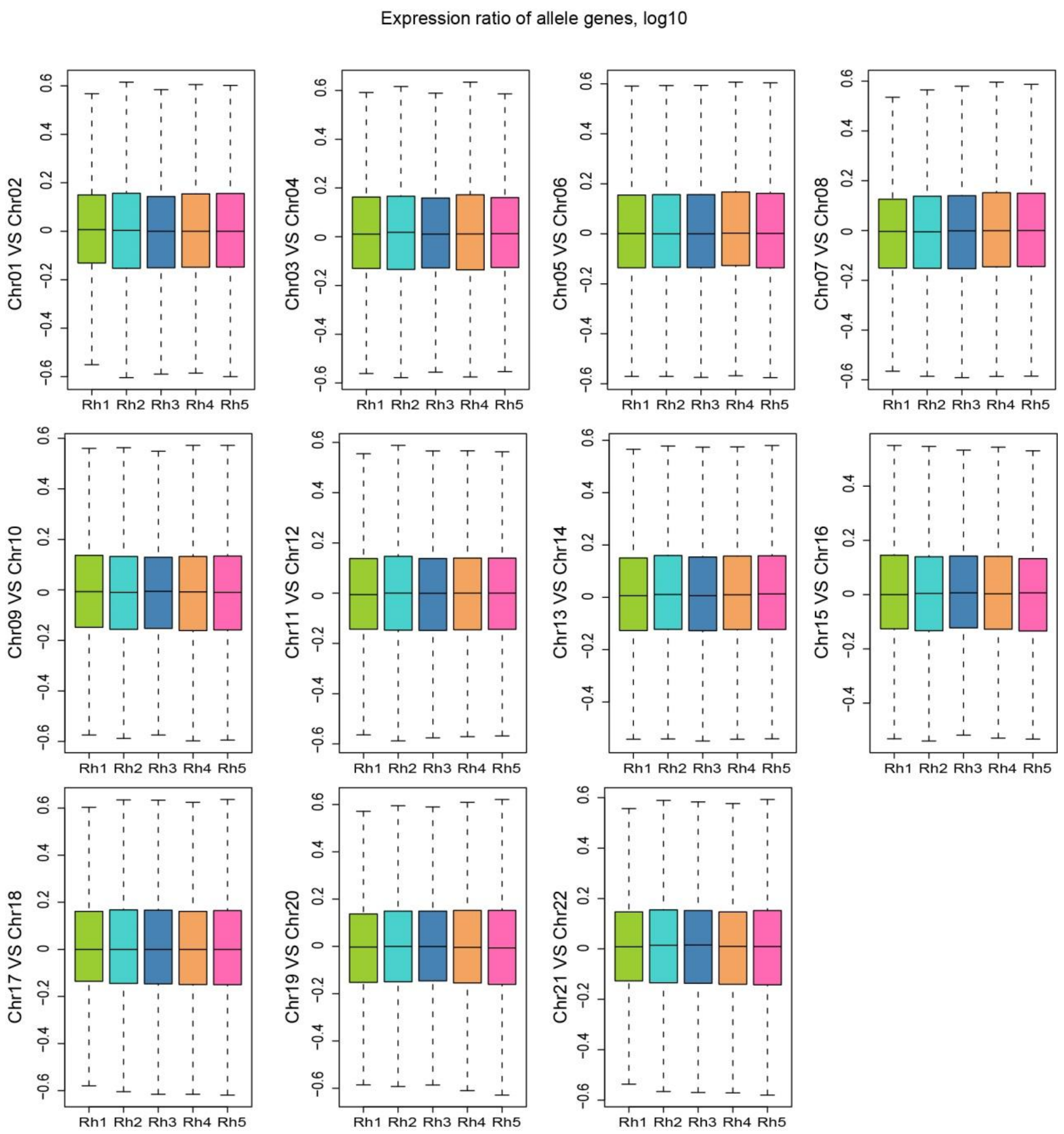

Supplementary Fig. S14 Ratio of the allelic gene expression in paired chromosomes of haplotype 0 and haplotype 1.
