# Supplementary material for: Haplotype-resolved genome of diploid ginger (Zingiber officinale) and its unique gingerol biosynthetic pathway
Source: Hortic Res. 2021 Aug 5;8:189. doi: 10.1038/s41438-021-00627-7 (PMC8342499; doi:10.1038/s41438-021-00627-7)
Supplement: Supplementary file 18 — Supplementary Fig. S17 [file 41438_2021_627_MOESM18_ESM.pdf]

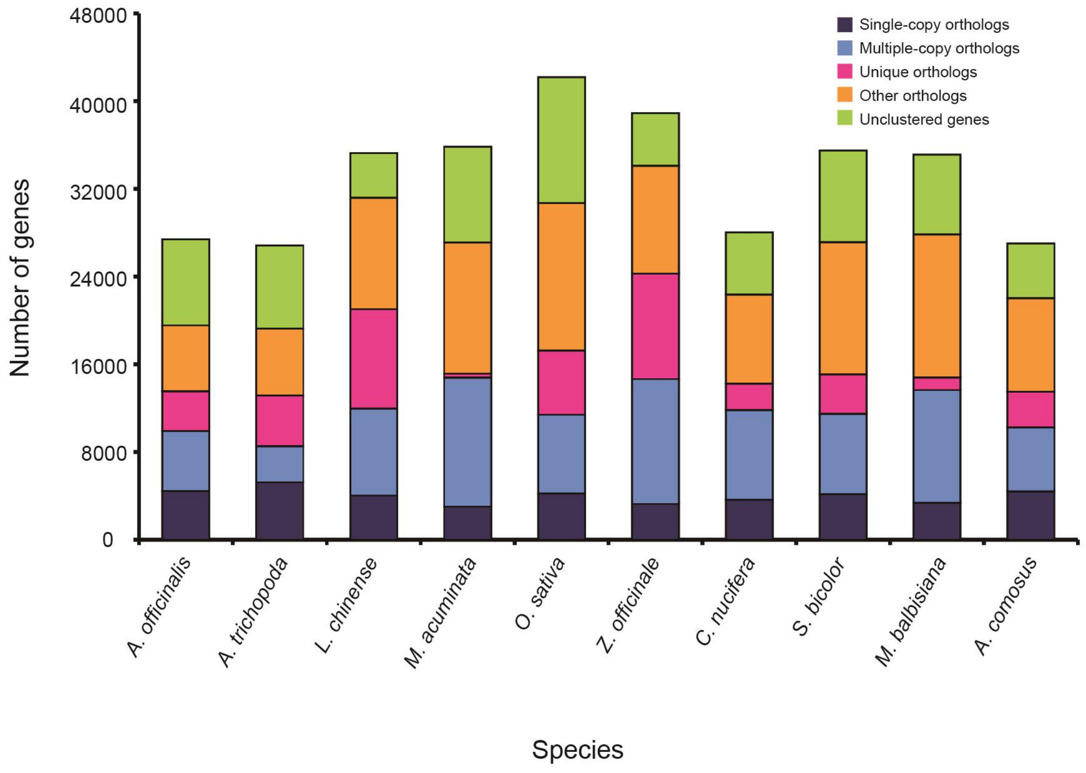

Supplementary Fig. S17 Number of genes in each category that were defined by OrthoMCL. Single-copy orthologs, multiple-copy orthologs, unique papalogs, other orthologs and unclustered genes were analyzed here.
