# Supplementary material for: Haplotype-resolved genome of diploid ginger (Zingiber officinale) and its unique gingerol biosynthetic pathway
Source: Hortic Res. 2021 Aug 5;8:189. doi: 10.1038/s41438-021-00627-7 (PMC8342499; doi:10.1038/s41438-021-00627-7)
Supplement: Supplementary file 20 — Supplementary Fig. S19 [file 41438_2021_627_MOESM20_ESM.pdf]

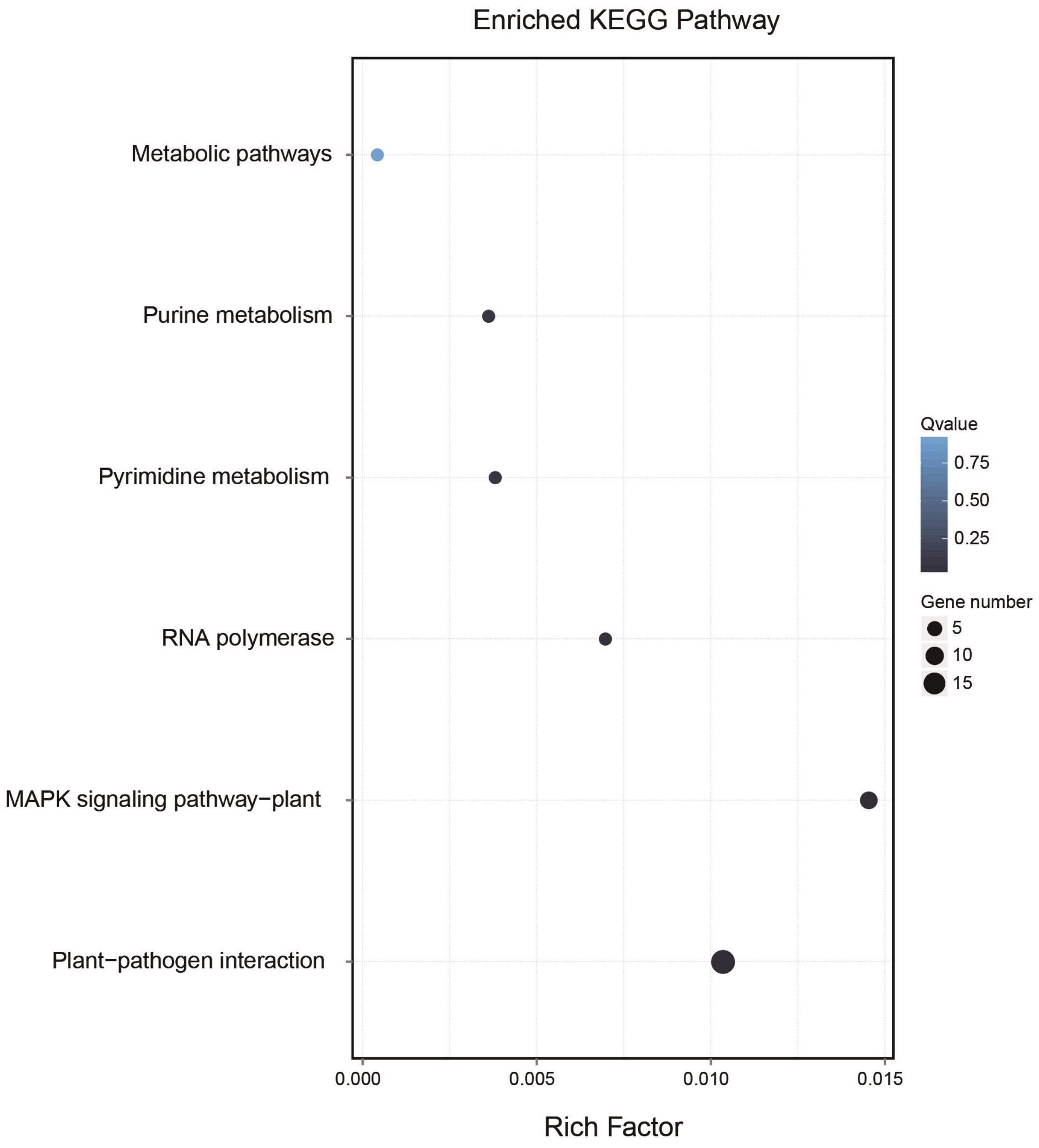

Supplementary Fig. S19 KEGG enrichment of ginger contraction genes. The color gradient stands for the Qvalue and the dot size represents the gene number.
