# Supplementary material for: Haplotype-resolved genome of diploid ginger (Zingiber officinale) and its unique gingerol biosynthetic pathway
Source: Hortic Res. 2021 Aug 5;8:189. doi: 10.1038/s41438-021-00627-7 (PMC8342499; doi:10.1038/s41438-021-00627-7)
Supplement: Supplementary file 23 — Supplementary Fig. S22 [file 41438_2021_627_MOESM23_ESM.pdf]

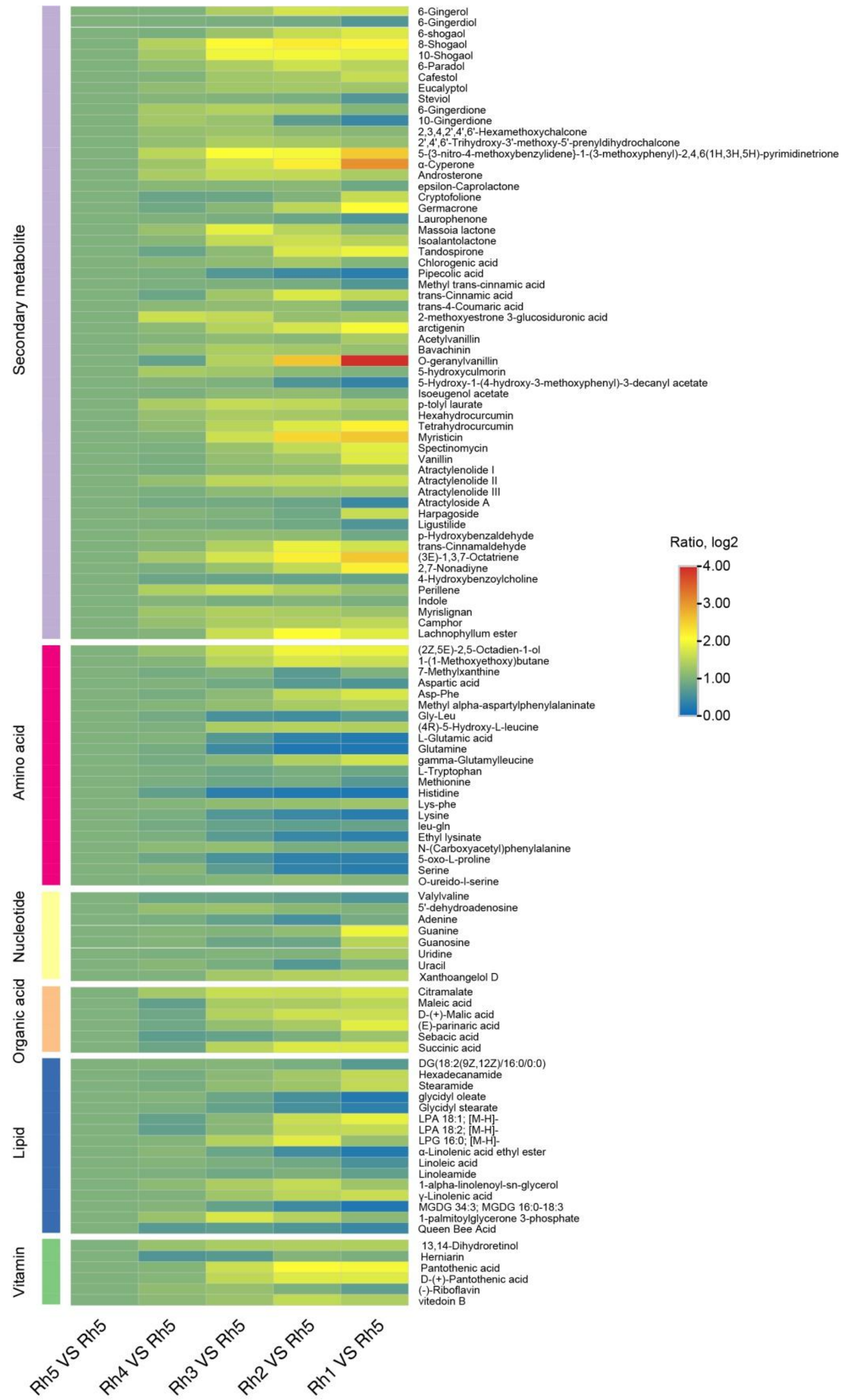

Supplementary Fig. S22 The concentration tendency of the identified metabolites in five rhizome developmental stages. The horizontal axis represents the ratio of metabolite abundance in each stage (Rh1-5) to Rh5.
