# Supplementary material for: Haplotype-resolved genome of diploid ginger (Zingiber officinale) and its unique gingerol biosynthetic pathway
Source: Hortic Res. 2021 Aug 5;8:189. doi: 10.1038/s41438-021-00627-7 (PMC8342499; doi:10.1038/s41438-021-00627-7)
Supplement: Supplementary file 25 — Supplementary Fig. S24 [file 41438_2021_627_MOESM25_ESM.pdf]

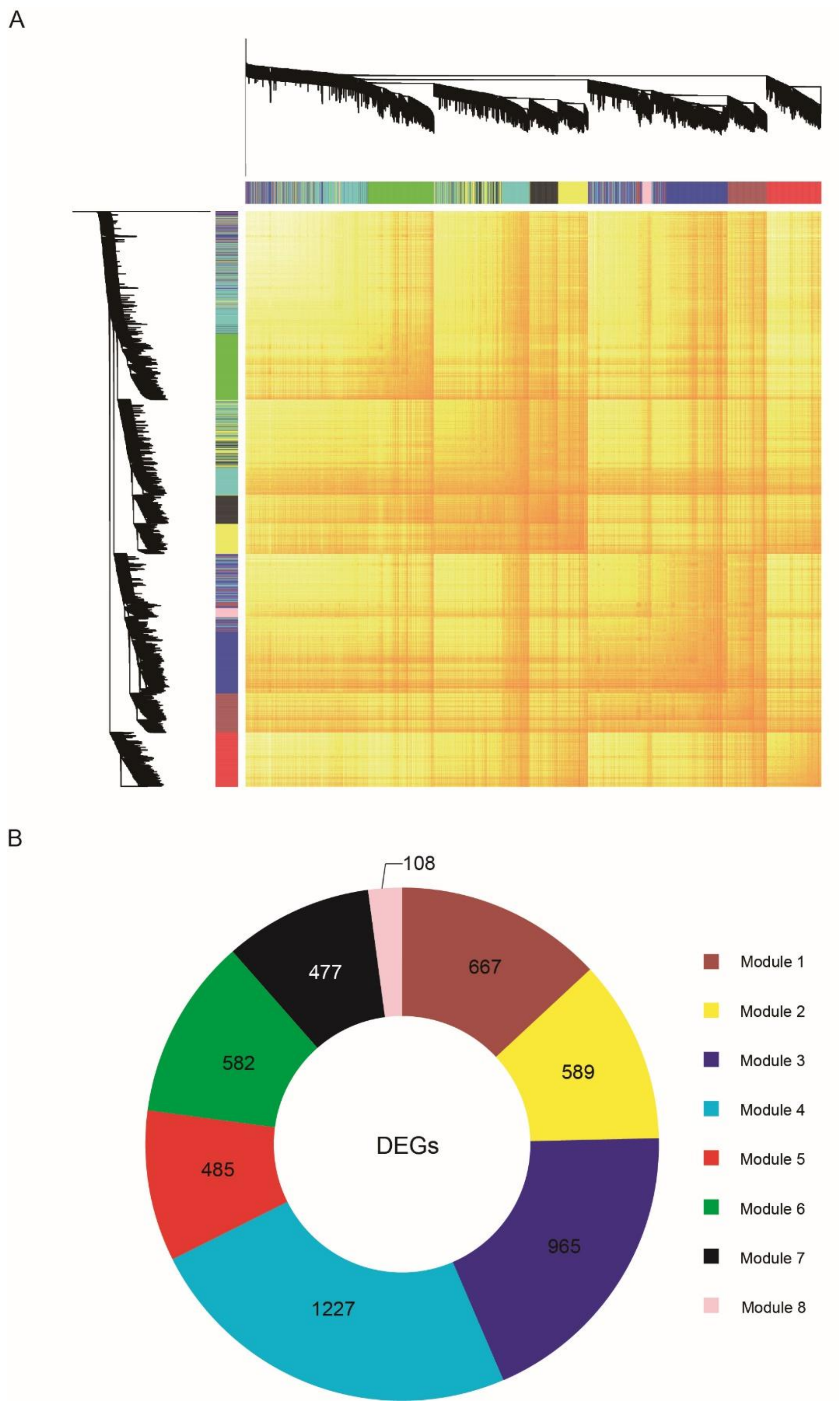


**Supplementary Fig. S24** Gene co-expression among the five rhizome developmental stages**.** (A) Heat maps depicting DEGs in rhizome were organized among 8 modules. (B) DEGs are non-randomly distributed across 8 modules. The different colors of the regions correspond to the different modules.
