# Supplementary material for: Haplotype-resolved genome of diploid ginger (Zingiber officinale) and its unique gingerol biosynthetic pathway
Source: Hortic Res. 2021 Aug 5;8:189. doi: 10.1038/s41438-021-00627-7 (PMC8342499; doi:10.1038/s41438-021-00627-7)
Supplement: Supplementary file 26 — Supplementary Fig. S25 [file 41438_2021_627_MOESM26_ESM.pdf]

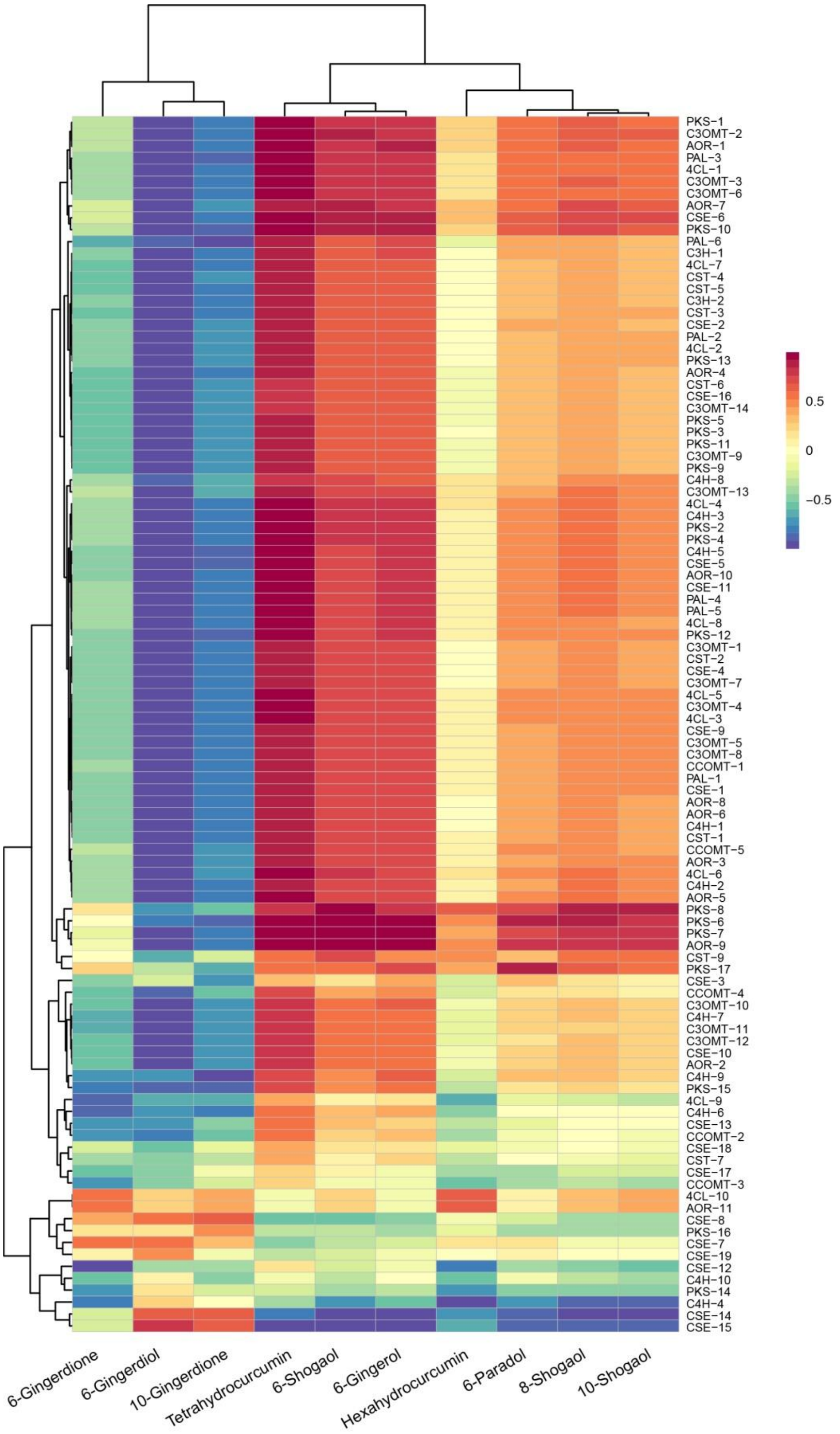

Supplementary Fig. S25 The association analyses between key genes and metabolites in the pathways of gingerol biosynthesis. The red and blue colours indicate the positive and negative correlation between metabolite and genes, respectively.
