# Supplementary material for: Haplotype-resolved genome of diploid ginger (Zingiber officinale) and its unique gingerol biosynthetic pathway
Source: Hortic Res. 2021 Aug 5;8:189. doi: 10.1038/s41438-021-00627-7 (PMC8342499; doi:10.1038/s41438-021-00627-7)
Supplement: Supplementary file 27 — Supplementary Fig. S26 [file 41438_2021_627_MOESM27_ESM.pdf]

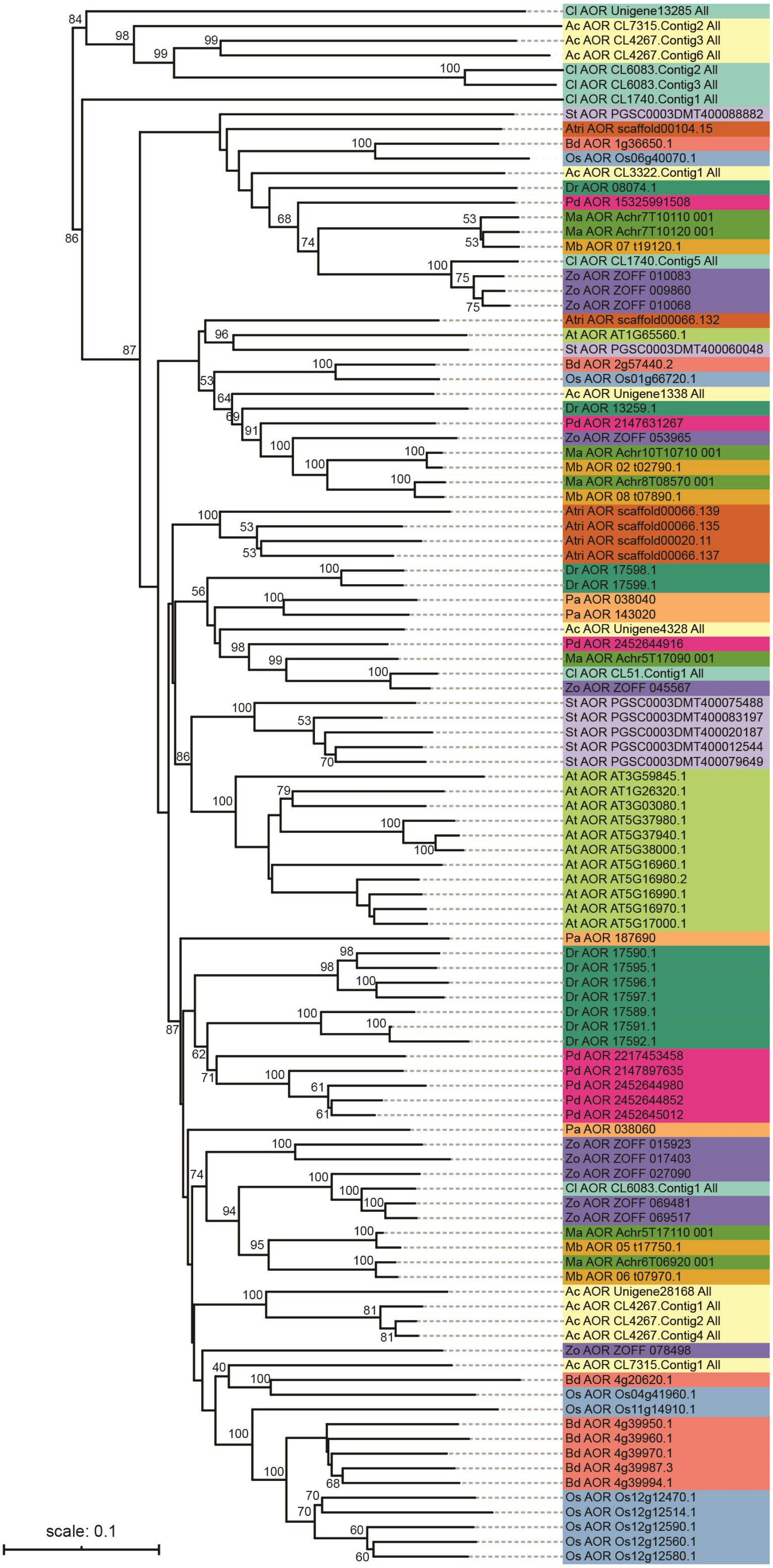

Cl AOR Unigene13285 All
Ac AOR CL7315.Contig2 All
Ac AOR CL4267.Contig3 All
Ac AOR CL4267.Contig6 All
Cl AOR CL6083.Contig2 All
Cl AOR CL6083.Contig3 All
Cl AOR CL1740.Contig1 All
St AOR PGSC0003DMT400088882
Atri AOR scaffold00104.15
Bd AOR 1g36650.1
Os AOR Os06g40070.1
Ac AOR CL3322.Contig1 All
Dr AOR 08074.1
Pd AOR 15325991508
Ma AOR Achr7T10110 001
Ma AOR Achr7T10120 001
Mb AOR 07 t19120.1
Cl AOR CL1740.Contig5 All
Zo AOR ZOFF 010083
Zo AOR ZOFF 009860
Zo AOR ZOFF 010068
Atri AOR scaffold00066.132
At AOR AT1G65560.1
St AOR PGSC0003DMT400060048
Bd AOR 2g57440.2
Os AOR Os01g66720.1
Ac AOR Unigene1338 All
Dr AOR 13259.1
Pd AOR 2147631267
Zo AOR ZOFF 053965
Ma AOR Achr10T10710 001
Mb AOR 02 t02790.1
Ma AOR Achr8T08570 001
Mb AOR 08 t07890.1
Atri AOR scaffold00066.139
Atri AOR scaffold00066.135
Atri AOR scaffold00020.11
Atri AOR scaffold00066.137
Dr AOR 17598.1
Dr AOR 17599.1
Pa AOR 038040
Pa AOR 143020
Ac AOR Unigene4328 All
Pd AOR 2452644916
Ma AOR Achr5T17090 001
Cl AOR CL51.Contig1 All
Zo AOR ZOFF 045567
St AOR PGSC0003DMT400075488
St AOR PGSC0003DMT400083197
St AOR PGSC0003DMT400020187
St AOR PGSC0003DMT400012544
St AOR PGSC0003DMT400079649
At AOR AT3G59845.1
At AOR AT1G26320.1
At AOR AT3G03080.1
At AOR AT5G37980.1
At AOR AT5G37940.1
At AOR AT5G38000.1
At AOR AT5G16960.1
At AOR AT5G16980.2
At AOR AT5G16990.1
At AOR AT5G16970.1
At AOR AT5G17000.1
Pa AOR 187690
Dr AOR 17590.1
Dr AOR 17595.1
Dr AOR 17596.1
Dr AOR 17597.1
Dr AOR 17589.1
Dr AOR 17591.1
Dr AOR 17592.1
Pd AOR 2217453458
Pd AOR 2147897635
Pd AOR 2452644980
Pd AOR 2452644852
Pd AOR 2452645012
Pa AOR 038060
Zo AOR ZOFF 015923
Zo AOR ZOFF 017403
Zo AOR ZOFF 027090
Cl AOR CL6083.Contig1 All
Zo AOR ZOFF 069481
Zo AOR ZOFF 069517
Ma AOR Achr5T17110 001
Mb AOR 05 t17750.1
Ma AOR Achr6T06920 001
Mb AOR 06 t07970.1
Ac AOR Unigene28168 All
Ac AOR CL4267.Contig1 All
Ac AOR CL4267.Contig2 All
Ac AOR CL4267.Contig4 All
Zo AOR ZOFF 078498
Ac AOR CL7315.Contig1 All
Bd AOR 4g20620.1
Os AOR Os04g41960.1
Os AOR Os11g14910.1
Bd AOR 4g39950.1
Bd AOR 4g39960.1
Bd AOR 4g39970.1
Bd AOR 4g39987.3
Bd AOR 4g39994.1
Os AOR Os12g12470.1
Os AOR Os12g12514.1
Os AOR Os12g12590.1
Os AOR Os12g12560.1
Os AOR Os12g12580.1
scale: 0.1

Supplementary Fig. S26 Phylogenetic analysis of the *AOR* gene family. The unrooted neighbor-joining (NJ) tree of 106 *AOR* genes in 13 plant species which include *B. distachyon*, *D. rotundata*, *P. aphrodite*, *P. dactylifera*, *A. calamus*, *C. longa*, *A. thaliana*, *S. tuberosum M. acuminate*, *M. balbisiana*, *O. sativa*, *Z. officinale*, and *A. trichopoda*. Bootstrap values were shown for each branch. Genes on branch ends from different species were denoted by different colors.
