# Supplementary material for: Haplotype-resolved genome of diploid ginger (Zingiber officinale) and its unique gingerol biosynthetic pathway
Source: Hortic Res. 2021 Aug 5;8:189. doi: 10.1038/s41438-021-00627-7 (PMC8342499; doi:10.1038/s41438-021-00627-7)
Supplement: Supplementary file 28 — Supplementary Fig. S27 [file 41438_2021_627_MOESM28_ESM.pdf]

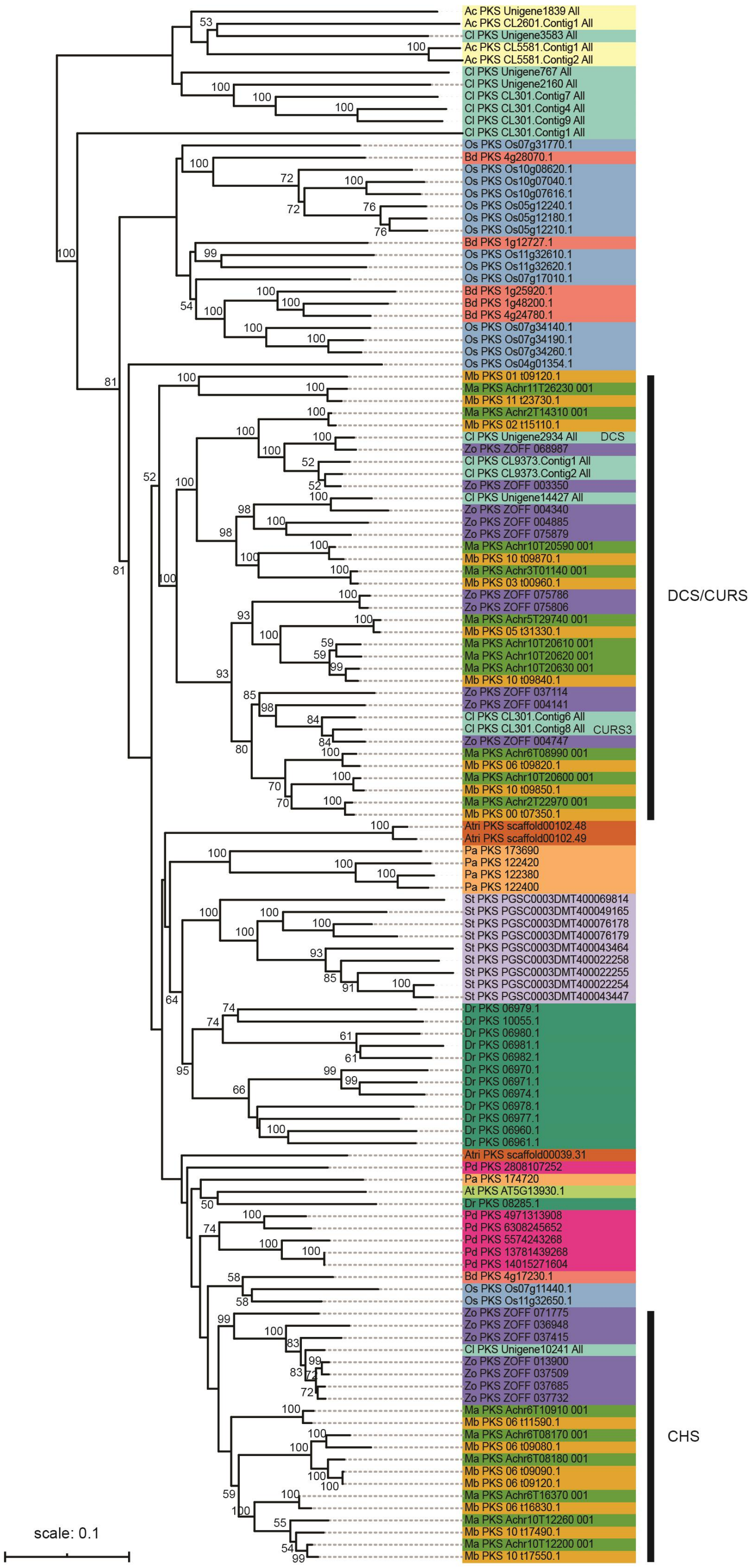
Ac PKS Unigene1839 All
Ac PKS CL2601.Contig1 All
Cl PKS Unigene3583 All
Ac PKS CL5581.Contig1 All
Ac PKS CL5581.Contig2 All
Cl PKS Unigene767 All
Cl PKS Unigene2160 All
Cl PKS CL301.Contig7 All
Cl PKS CL301.Contig4 All
Cl PKS CL301.Contig9 All
Cl PKS CL301.Contig1 All
Os PKS Os07g31770.1
Bd PKS 4g28070.1
Os PKS Os10g08620.1
Os PKS Os10g07040.1
Os PKS Os10g07616.1
Os PKS Os05g12240.1
Os PKS Os05g12180.1
Os PKS Os05g12210.1
Bd PKS 1g12727.1
Os PKS Os11g32610.1
Os PKS Os11g32620.1
Os PKS Os07g17010.1
Bd PKS 1g25920.1
Bd PKS 1g48200.1
Bd PKS 4g24780.1
Os PKS Os07g34140.1
Os PKS Os07g34190.1
Os PKS Os07g34260.1
Os PKS Os04g01354.1
Mb PKS 01 t09120.1
Ma PKS Achr11T26230 001
Mb PKS 11 t23730.1
Ma PKS Achr2T14310 001
Mb PKS 02 t15110.1
Cl PKS Unigene2934 All
DCS
Zo PKS ZOFF 068987
Cl PKS CL9373.Contig1 All
Cl PKS CL9373.Contig2 All
Zo PKS ZOFF 003350
Cl PKS Unigene14427 All
Zo PKS ZOFF 004340
Zo PKS ZOFF 004885
Zo PKS ZOFF 075879
Ma PKS Achr10T20590 001
Mb PKS 10 t09870.1
Ma PKS Achr3T01140 001
Mb PKS 03 t00960.1
Zo PKS ZOFF 075786
Zo PKS ZOFF 075806
Ma PKS Achr5T29740 001
Mb PKS 05 t31330.1
Ma PKS Achr10T20610 001
Ma PKS Achr10T20620 001
Ma PKS Achr10T20630 001
Mb PKS 10 t09840.1
Zo PKS ZOFF 037114
Zo PKS ZOFF 004141
Cl PKS CL301.Contig6 All
Cl PKS CL301.Contig8 All
CURS3
Zo PKS ZOFF 004747
Ma PKS Achr6T08990 001
Mb PKS 06 t09820.1
Ma PKS Achr10T20600 001
Mb PKS 10 t09850.1
Ma PKS Achr2T22970 001
Mb PKS 00 t07350.1
Atri PKS scaffold00102.48
Atri PKS scaffold00102.49
Pa PKS 173690
Pa PKS 122420
Pa PKS 122380
Pa PKS 122400
St PKS PGSC0003DMT400069814
St PKS PGSC0003DMT400049165
St PKS PGSC0003DMT400076178
St PKS PGSC0003DMT400076179
St PKS PGSC0003DMT400043464
St PKS PGSC0003DMT400022258
St PKS PGSC0003DMT400022255
St PKS PGSC0003DMT400022254
St PKS PGSC0003DMT400043447
Dr PKS 06979.1
Dr PKS 10055.1
Dr PKS 06980.1
Dr PKS 06981.1
Dr PKS 06982.1
Dr PKS 06970.1
Dr PKS 06971.1
Dr PKS 06974.1
Dr PKS 06978.1
Dr PKS 06977.1
Dr PKS 06960.1
Dr PKS 06961.1
Atri PKS scaffold00039.31
Pd PKS 2808107252
Pa PKS 174720
At PKS AT5G13930.1
Dr PKS 08285.1
Pd PKS 4971313908
Pd PKS 6308245652
Pd PKS 5574243268
Pd PKS 13781439268
Pd PKS 14015271604
Bd PKS 4g17230.1
Os PKS Os07g11440.1
Os PKS Os11g32650.1
Zo PKS ZOFF 071775
Zo PKS ZOFF 036948
Zo PKS ZOFF 037415
Cl PKS Unigene10241 All
Zo PKS ZOFF 013900
Zo PKS ZOFF 037509
Zo PKS ZOFF 037685
Zo PKS ZOFF 037732
Ma PKS Achr6T10910 001
Mb PKS 06 t11590.1
Ma PKS Achr6T08170 001
Mb PKS 06 t09080.1
Ma PKS Achr6T08180 001
Mb PKS 06 t09090.1
Mb PKS 06 t09120.1
Ma PKS Achr6T16370 001
Mb PKS 06 t16830.1
Ma PKS Achr10T12260 001
Mb PKS 10 t17490.1
Ma PKS Achr10T12200 001
Mb PKS 10 t17550.1
DCS/CURS
CHS
scale: 0.1

Supplementary Fig. S27 Phylogenetic analysis of the *PKS* gene family. The unrooted neighbor-joining (NJ) tree of 128 *PKS* genes in 13 plant species which include Phylogenetic analysis of the *AOR* gene family. The unrooted neighbor-joining (NJ) tree of 106 *AOR* genes in 13 plant species which include *B. distachyon*, *D. rotundata*, *P. aphrodite*, *P. dactylifera*, *A. calamus*, *C. longa*, *A. thaliana*, *S. tuberosum M. acuminate*, *M. balbisiana*, *O. sativa*, *Z. officinale*, and *A. trichopoda*. Bootstrap values were shown for each branch. Genes on branch ends from different species were denoted by different colors. The black bar indicated the unique subfamilies in gingerols biosynthesis pathway.
